# Structural and evolutionary adaptation of rhoptry kinases and pseudokinases, a family of coccidian virulence factors

**DOI:** 10.1186/1471-2148-13-117

**Published:** 2013-06-06

**Authors:** Eric Talevich, Natarajan Kannan

**Affiliations:** 1 Institute of Bioinformatics, University of Georgia, Athens, Georgia, USA; 2 Department of Biochemistry, University of Georgia, Athens, Georgia, USA

## Abstract

**Background:**

The widespread protozoan parasite *Toxoplasma gondii* interferes with host cell functions by exporting the contents of a unique apical organelle, the rhoptry. Among the mix of secreted proteins are an expanded, lineage-specific family of protein kinases termed rhoptry kinases (ROPKs), several of which have been shown to be key virulence factors, including the pseudokinase ROP5. The extent and details of the diversification of this protein family are poorly understood.

**Results:**

In this study, we comprehensively catalogued the ROPK family in the genomes of *Toxoplasma gondii*, *Neospora caninum* and *Eimeria tenella*, as well as portions of the unfinished genome of *Sarcocystis neurona*, and classified the identified genes into 42 distinct subfamilies. We systematically compared the rhoptry kinase protein sequences and structures to each other and to the broader superfamily of eukaryotic protein kinases to study the patterns of diversification and neofunctionalization in the ROPK family and its subfamilies. We identified three ROPK sub-clades of particular interest: those bearing a structurally conserved N-terminal extension to the kinase domain (NTE), an *E. tenella*-specific expansion, and a basal cluster including ROP35 and BPK1 that we term ROPKL. Structural analysis in light of the solved structures ROP2, ROP5, ROP8 and in comparison to typical eukaryotic protein kinases revealed ROPK-specific conservation patterns in two key regions of the kinase domain, surrounding a ROPK-conserved insert in the kinase hinge region and a disulfide bridge in the kinase substrate-binding lobe. We also examined conservation patterns specific to the NTE-bearing clade. We discuss the possible functional consequences of each.

**Conclusions:**

Our work sheds light on several important but previously unrecognized features shared among rhoptry kinases, as well as the essential differences between active and degenerate protein kinases. We identify the most distinctive ROPK-specific features conserved across both active kinases and pseudokinases, and discuss these in terms of sequence motifs, evolutionary context, structural impact and potential functional relevance.

By characterizing the proteins that enable these parasites to invade the host cell and co-opt its signaling mechanisms, we provide guidance on potential therapeutic targets for the diseases caused by coccidian parasites.

## Background

*Toxoplasma gondii* is an intracellular parasite that infects a wide range of hosts, including an estimated one-third of the world’s human population [1]. The resulting disease toxoplasmosis can be serious in pregnant women and immunocompromised individuals, and as an opportunistic infection associated with AIDS and cancer patients [2]. *T. gondii* and its evolutionary relatives, the Coccidia, form a clade of parasitic protozoa involved in many human and veterinary diseases such as toxoplasmosis and coccidiosis. Coccidians are a lineage within the protozoan phylum Apicomplexa, which also includes the deadly malaria pathogen *Plasmodium falciparum*. Thus, *T. gondii* also serves as an experimentally tractable model organism for studying the shared and contrasting biological properties of the Apicomplexa and other intracellular parasites [3,4].

Apicomplexans contain a unique system of apical organelles called the apical complex, consisting of rhoptries, micronemes and dense granules [5]. At the initiation of host cell invasion, the contents of the rhoptries are injected into the host cell and the forming parasitophorous vacuole which protects the intracellular parasite [6]. Once there, the parasite proteins can disrupt host cell signaling and defense mechanisms and assist in recruiting host organelles [7].

Proteomic profiling of *T. gondii* rhoptries [8] and analyis of apicomplexan genomic sequences [9-12] revealed that many of the proteins secreted by coccidians are protein kinases, a class of enzymes that regulate cell signal transduction through phosphorylation. This expanded, rapidly evolving family of kinases and pseudokinases has been termed the rhoptry kinase (ROPK) family [10], or ROP2 family, in reference to a representative member of the family [9]. While rhoptry kinases appear to be unique to the Coccidia, the involvement of lineage-specific protein kinase families in host-parasite interactions is observed across the Apicomplexa [13]. Several rhoptry kinases have been shown to be involved in virulence and alteration of host cell transcription [7,14]. These include ROP18, a key modulator of parasite growth and virulence which is localized to the parasitophorous vacuole membrane (PVM) [15,16], and ROP5, another PVM-associated protein which assists ROP18 in blocking the host immune response [17-21]. ROP16 localizes to the host cell nucleus and interacts with the STAT3 and STAT6 immune-response signaling pathways [22-26], and ROP38 has been implicated in the modulation of host MAPK signaling [10].

Protein kinases are a diverse family of enzymes which have been successfully targeted for inhibition in human cancers, and show promise for treating infections by protozoan pathogens as well [27]. ATP-competitive small-molecule inhibitors have been developed to specifically target catalytically active protein kinases in parasitic protozoa [28]. Since many of the ROPKs appear to also be catalytically active, there may be an opportunity to target these kinases for infectious diseases. However, the “catalytic triad” of residues considered essential for kinase enzymatic activity [29] is altered in about half of the identified ROPKs [10]. Pseudokinases have been observed to perform important functions in other systems, typically through inducing allosteric changes in other interacting partners (e.g. [30,31]; reviewed in [32-34]). The overall expansion of pseudokinases in the ROPK family underscores observations that some catalytically inactive ROPKs nonetheless play important, functional roles through interaction with other proteins [18,19,35]. Structural studies showed that the pseudokinase virulence factors ROP2, ROP8 and ROP5 do indeed form a protein kinase fold; ROP2 and ROP8 were indicated to be unable to bind ATP [36], while ROP5 was shown to bind ATP in an atypical, noncatalytic conformation [37]. An interplay between ROP5, the active kinase ROP18 and a host immunity-related GTPase has been identified [18,19], demonstrating the potential for complex interplay between rhoptry kinases and the host cell signaling pathways. However, the full extent of the diversity in ROPK family, in terms of function, potential interacting partners, protein structure and structural mechanisms, is poorly understood. With the availability of molecular sequence and structural data from multiple strains of *T. gondii* and related apicomplexans, we can use comparative methods to examine the molecular evolution of ROPKs and identify functional shifts that may point to distinct regulatory roles and mechanisms.

We catalogued the rhoptry kinases in several fully sequenced coccidian genomes, including *Toxoplasma gondii*, *Neospora caninum*, *Sarcocystis neurona* and *Eimeria tenella*, and compared them to the broader eukaryotic protein kinase (ePK) superfamily and to each other to study the patterns of diversification and neofunctionalization in the ROPK family and its subfamilies. We propose previously unidentified rhoptry kinases in each of these genomes, including several putative new ROPK subfamilies. We studied the variation in these subfamilies in light of the solved structures of ROP2, ROP8 and ROP5 proteins, and relative to “typical” eukaryotic protein kinases. Both pseudokinases and catalytically active kinases appear to be prevalent throughout the ROPK family. We found a striking co-evolution of structural inserts within the canonical protein kinase domain and the residues that interact with them. Most noteworthy among these is a pattern of residues surrounding the ROPK-specific *α*C’ helix in the kinase “hinge” region. We also recovered another pattern of co-conserved cysteines that form a disulfide bond in the substrate-binding C-lobe. We then discuss some possible functional consequences of these distinguishing features of the ROPK family.

## Results

To examine the molecular evolution and functional shifts in ROPKs, we used the genomic, mRNA and proteomic sequences of multiple *T. gondii* strains, *Neospora caninum*, *Sarcocystis neurona* and *Eimeria tenella* to develop profiles for 42 subfamilies of ROPK, reflecting orthology as well as chromosomal patterns of tandem repeats (see Methods).

We used these sequence profiles to perform an analysis of evolutionary constraints, applying statistical tests of contrasting conservation between gene clades to identify potential sites of subfunctionalization and neofunctionalization in the ROPK family and each ROPK subfamily. We then mapped the sites and regions of interest onto solved structures of ROP2, ROP8 and ROP5 to examine the structural and possible functional roles these features may play within the parasite proteins.

### Global trends in the ROPK family

We used a set of HMM profiles derived from our subfamily sequence alignments to scan the translated gene model sequences available for *T. gondii* strains GT1, ME49 and VEG, *N. caninum* and *E. tenella* and classify putative ROPK genes into the identified subfamilies. We found 37, 55 and 38 ROPK genes in *T. gondii* strains GT1, ME49 and VEG, respectively, 44 in *N. caninum* and 27 in *E. tenella* (Additional file 1). The elevated ROPK counts in *T. gondii* ME49 relative to the other strains is probably due to differences in sequencing depth and the quality of assembly and gene model annotation; we also found genomic evidence of unannotated orthologs in the other strains. As suggested by Reese and Boyle [35], ROPK genes are often present in expanded loci (sites of gene duplication, usually in tandem array) and are probably undercounted in annotated genomes.

By incorporating sequences from multiple coccidian species into HMM profiles, we were able to identify several putative ROPKs that were not identified in previous computational surveys [9,10]. These include the proposed subfamilies ROP47, ROP48, ROP49 and ROP50, present in *T. gondii* and *N. caninum*, and the *E. tenella*-specific subfamilies ROPK-Eten1, ROPK-Eten2a, ROPK-Eten2b, ROPK-Eten3, ROPK-Eten4, ROPK-Eten5 and ROPK-Eten6. We suggest these to be likely rhoptry kinases on the basis of sequence homology, phylogenetic placement, signal peptide presence, and existing experimental evidence. Protein or mRNA expression has been previously observed for at least one member of each of these proposed subfamilies, indicating that they are not pseudogenes. ROP47, ROP49 and ROP50 are predicted to contain a signal peptide. The gene coding for ROP48 has only been annotated in *T. gondii* strain ME49 (TGME49_234950, numbered TGME49_034950 in ToxoDB prior to version 8.0), but we identified genomic regions with 95% sequence identity to this protein sequence on chromosome X of strains VEG and GT1 as well. Recently, a proteomics study observed two *E. tenella* proteins expressed during the sporozoite stage and localized in the rhoptries: ETH_00027700, which we assigned to the ROPK-Eten1 subfamily, and ETH_00005190, which we assigned to the ROPK-Unique category [38]. A search of the available *S. neurona* ESTs and genomic scaffolds indicates that ROPKs are prevalent in this species as well, though we cannot assign a specific number until the assembly is complete. The subfamilies that have clear representatives in all four of the surveyed species are ROP21/27 and ROP35.

In *S. neurona*, rhoptries are present in the sporozoite [39] and bradyzoite [40] stages but absent from schizonts and merozoites [41]. Surprisingly, we found *S. neurona* genomic regions and expressed sequence tags (ESTs) from the schizont and merozoite stages that appear to code for rhoptry kinases. Of the ESTs currently available in the NCBI GenBank EST database, we identified seven putative rhoptry kinases [GenBank:BM303139.1, BM303688.1, BQ749596.1, BQ750005.1, BU085181.1, CO748650.1, CV193082.1], all obtained from the *S. neurona* merozoite stage, evidence that these genes are indeed expressed despite the absence of rhoptry organelles during this life stage. We also examined genomic open reading frames (ORFs) for signal peptides using the program SignalP [42] and identified likely signal peptide regions and cleavage sites in several of the ORFs that we predicted to encode rhoptry kinases, suggesting that at least some of these are likely to be exported.

Both pseudokinases and catalytically active kinases appear to be prevalent throughout the ROPK family, in roughly equal numbers of subfamilies. The pseudokinase subfamilies are distributed throughout the phylogenetic tree, rather than forming any distinct clade, suggesting that the evolutionary pressures that lead to the degeneration of paralogs into pseudokinases have applied throughout the ROPK family.

#### Phylogenetic clustering reveals distinct sub-clades

**Figure 1:**
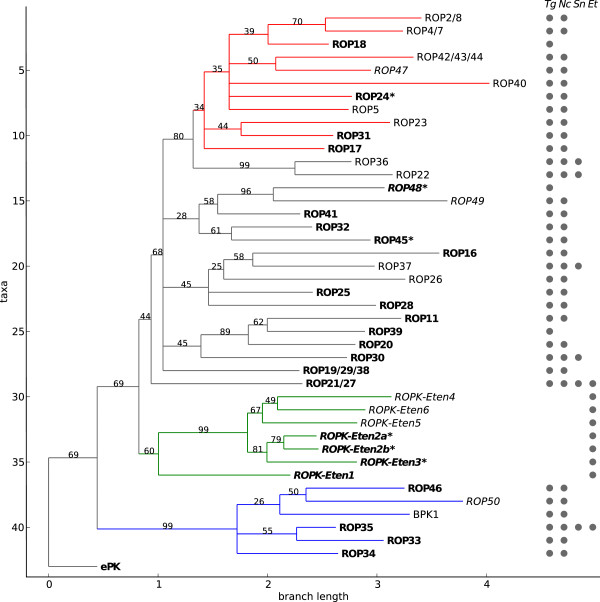
**Phylogeny of rhoptry kinase subfamilies.** Predicted or known active kinases are labeled in bold text, and kinases that may have a noncanonical catalytic mechanism are marked with an asterisk. Newly proposed ROPK subfamilies are labeled in italic text. The clade indicated in red contains the ROPK subfamilies with a homologous N-terminal extension to the kinase domain (NTE). The clade in green is specific to *E. tenella*. The divergent “ROPKL” clade is shown in blue. Branch labels indicate bootstrap support. The grid along the right side indicates the species in which each subfamily appears: *T. gondii* (Tg), *N. caninum* (Nc), *S. neurona* (Sn) and *E. tenella* (Et).

We inferred a phylogenetic tree from the consensus sequences of each of the ROPK subfamilies to illustrate evolutionary patterns within the ROPK family (Figure 1). Several distinct clades emerged, which we examined more specifically: rhoptry kinases with homology to the N-terminal extension (NTE) observed in ROP2, ROP8 and ROP5 structures (discussed below); an expanded clade of seven subfamilies specific to *E. tenella*; and a basal clade of divergent, ROPK-like protein kinases, including ROP35 and BPK1, which we refer to as ROPKL here.

Within the *E. tenella*-specific clade, the putative ROPK proteins ETH_00028855, ETH_00020620 and ETH_00000075, which we placed in the subfamilies Eten2b, Eten3 and Eten4, respectively, were recently observed to be expressed solely in merozoites [38]. The emergence of this gene clade reflects the significant phylogenetic and phenotypic divergence of the oocyst-forming *E. tenella* from the other tissue-cyst-forming coccidian species we have examined here [43]. *E. tenella* also contains several putative ROPKs outside this clade, more closely related to the ROPKs found in *T. gondii* and *N. caninum*, which we placed in the ROPK-Unique category (Additional file 1).

The previously identified proteins in the ROPKL clade are ROP33, ROP34, ROP35 and ROP46. The clade also contains the brazyzoite-expressed pseudokinase BPK1 [44]. The gene models of the ROPKL proteins in *T. gondii* ME49, the best-annotated strain, all contain at least one intron, in contrast to most other ROPK genes, which are typically encoded by a single exon.

#### Known or likely catalytic kinases

In our analysis, we consider the catalytically essential residues to be the aspartate in the catalytic loop (“HRD” motif, D166^PKA^) and the aspartate in the Mg-binding loop at the start of the activation segment (“DFG” motif, D184^PKA^); we categorize the ROPK subfamilies missing either of these residues as pseudokinases. Additionally important residues involved in ATP positioning or conformational changes necessary for catalytic activity include a glycine in subdomain I (G52^PKA^), lysine in subdomain II (“VAIK” motif, K72^PKA^), glutamate in subdomain III (E91^PKA^) and asparagine in the catalytic loop (N171^PKA^) [29,45,46], as well as the F-helix aspartate which positions the catalytic loop (“DxW” motif, D220^PKA^) [47]. While catalysis has been observed in kinases that lack one or more of these residues, their absence usually indicates a noncanonical mechanism or impairment of activity [31,48,49].

**Figure 2:**
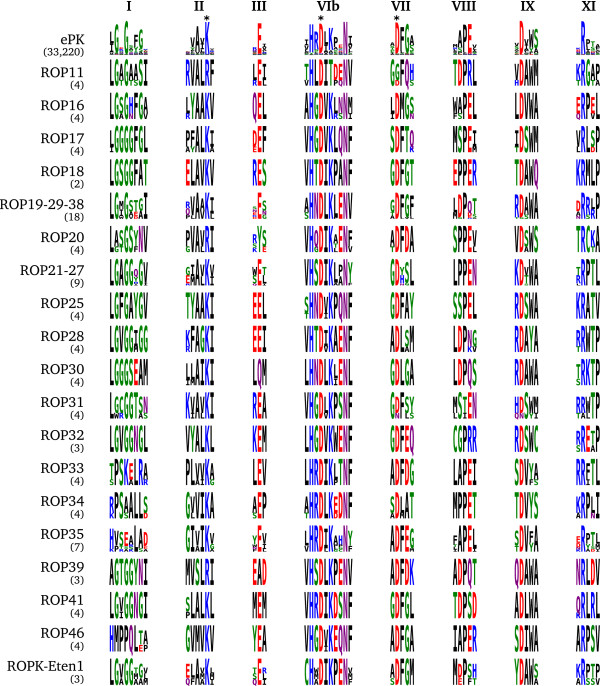
**Conserved motifs of catalytically active rhoptry kinase subfamilies.** Sequence logos of key regions in the kinase domain of the broader ePK superfamily and of predicted active ROPK subfamilies as they occur in the coccidian species examined. Letter height at each sequence position indicates greater conservation of that character in a multiple sequence alignment of a large set of ePK sequences (first row) and the annoted genomic sequences of each ROPK subfamily. Row labels indicate subfamily names, with the number of sequences in each alignment shown in parentheses. The ePK-conserved motifs shown are the glycine-rich loop in subdomain I, catalytic lysine in subdomain II, *α*C glutamate in subdomain III, catalytic loop in subdomain VIb, “DFG” in subdomain VII, “APE” in subdomain VIII, *α*F “DxW” in subdomain IX, and arginine in subdomain XI. The adjacent sequence sites surrounding each motif are included for context. Asterisks indicate above ePK motifs indicate the catalytic triad. Generated using the WebLogo [82] and ReportLab [83] libraries.

The subfamilies ROP11, ROP16, ROP17, ROP18, ROP19/29/38, ROP20, ROP21/27, ROP25, ROP28, ROP30, ROP31, ROP32, ROP35, ROP39 and ROP41 were previously suggested to be active kinases based on the conserved catalytic triad [10]. Phosphoryl transfer has been demonstrated experimentally for ROP18 [50] and ROP16 [24], and molecular modelling simulations have shown that ATP could dock in a typical conformation to ROP11, ROP16, ROP17 and ROP18 [36]. Our analysis additionally found the catalytically essential residues conserved in ROP33, ROP34 and ROP46, suggesting these may also be active kinases. Of the *E. tenella*-specific subfamilies we identified, ROPK-Eten1 also retains all of the key residues needed for catalysis (Figure 2).

#### Known or likely pseudokinases

**Figure 3:**
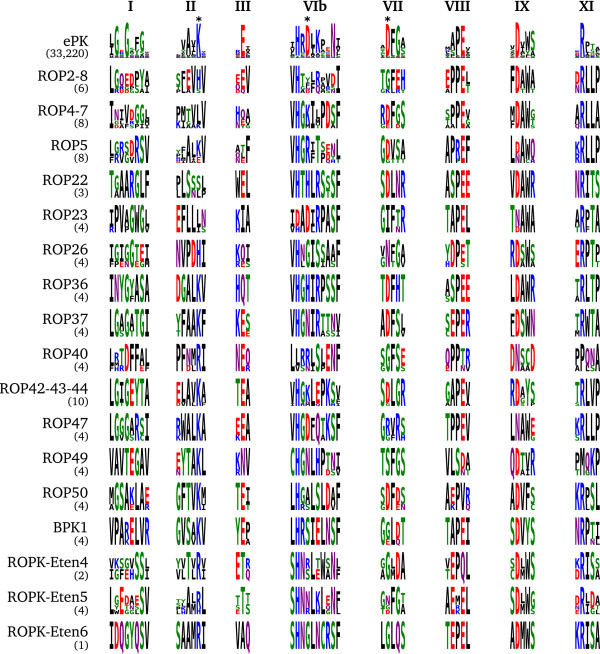
**Conserved motifs of likely inactive rhoptry kinase subfamilies.** Sequence logos of conserved motif regions in the kinase domain of the broader ePK superfamily and of predicted pseudokinase ROPK subfamilies as they occur in the coccidian species examined.

Kinases that lack one or more of the residues necessary for catalysis are likely to be non-catalytic pseudokinases. The apparent pseudokinase ROPK subfamilies are ROP2/8, ROP4/7, ROP5, ROP22, ROP23, ROP26, ROP36, ROP37, ROP40 and ROP42/43/44, as identified previously [10]. We include BPK1, previously noted as a *T. gondii* brazyzoite-expressed pseudokinase [44], in the ROPK family based on sequence similarity. Additionally, our proposed subfamilies ROP47, ROP49, ROP50, and the *E. tenella*-specific ROPK-Eten4, ROPK-Eten5 and ROPK-Eten6, are also missing key aspartates involved in the kinase catalytic mechanism and are likely to be pseudokinases (Figure 3). ROP50 does have an aspartate at the HRD+3 position (Figure 3), so in absence of a structure we cannot rule out that this nearby residue may play a compensatory role in catalysis.

Several of these pseudokinase subfamilies share the unusual characteristic of replacing the catalytic aspartate (in the kinase-conserved “HRD” motif) with a basic residue: ROP4/7 (HGK), ROP5 (HG[R/K/H]), ROP22 (HTH), ROP36 (HGH), ROP40 (LRR) and ROP42–43-44 (HGK), as previously noted [37].

#### Noncanonical kinases

**Figure 4:**
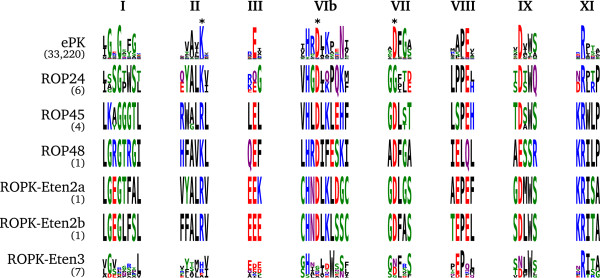
**Conserved motifs of ROPK subfamilies with potentially noncanonical catalytic mechanisms.** Sequence logos of conserved motif regions in the kinase domain of the broader ePK superfamily and of ROPK subfamilies with predicted noncanonical catalytic mechanisms as they occur in the coccidian species examined.

The subfamilies ROP24, ROP45 and the proposed ROP48, ROPK-Eten2a and ROPK-Eten2b have most of the residues necessary for catalysis, but with some differences in other typically conserved residues that suggest the mechanisms may be noncanonical (Figure 4).

In most active ePKs, an asparagine in the catalytic loop (N171^PKA^) coordinates a magnesium ion to position ATP in the active site [29]. This residue varies among some ROPKs: In ROP24, ROP45 and ROP48, the asparagine is replaced by a basic residue (lysine, histidine and lysine, respectively). The closely related *E. tenella*-specific subfamilies ROPK-Eten2a and ROPK-Eten2b have the catalytic loop motifs HNDLKLDG and HNDLKLSS, respectively, each replacing the ePK-conserved asparagine with a different residue type. Such replacements are rare in catalytically active kinases; in an alignment of ePK sequences (not shown), we observed only two cases in which the “HRD” motif is conserved without the accomanying asparagine, both of which have been shown to have noncanonical catalytic mechanisms: CASK [49], which replaces the asparagine with a cysteine, and Type II PAK [51], which has a serine.

The ePK-conserved lysine in subdomain II (*β*3) is replaced with arginine in ROP45, ROPK-Eten2a and ROPK-Eten2b, though the conserved C-helix glutamate is retained, suggesting the necessary salt bridge could still form in the active state of these kinase as in other ePKs. In ROP24, however, the lysine is retained but the corresponding C-helix glutamate is instead alanine, precluding a salt bridge. The DFG motif is replaced with the sequence GFT, though a potentially compensatory acidic residue appears at the DFG+1 position. These observations suggest that the activation mechanism [52,53] in ROP24 could be different from that of other ePKs. ROP48 retains the *β*3 lysine, *α*C glutamate and DFG motif; however, the substrate-binding lobe is quite divergent, with a dramatically shortened activation loop and F-helix, and the F-helix DxW motif is replaced with ESS, which suggests that the positioning of the catalytic loop occurs differently from other ePKs.

The *E. tenella*-specific subfamily ROPK-Eten3, in contrast to all the other identified ROPK subfamilies, appears to comprise both active and inactive kinases. The locus appears as a tandem repeat of 5 similar genes, with pairwise identity ranging from 32% to 52% (mean 41%), only one of which (ETH_00020585) retains the key residues indicating catalytic function (Figure 4).

### ROPK-conserved inserts within the protein kinase domain

**Figure 5:**
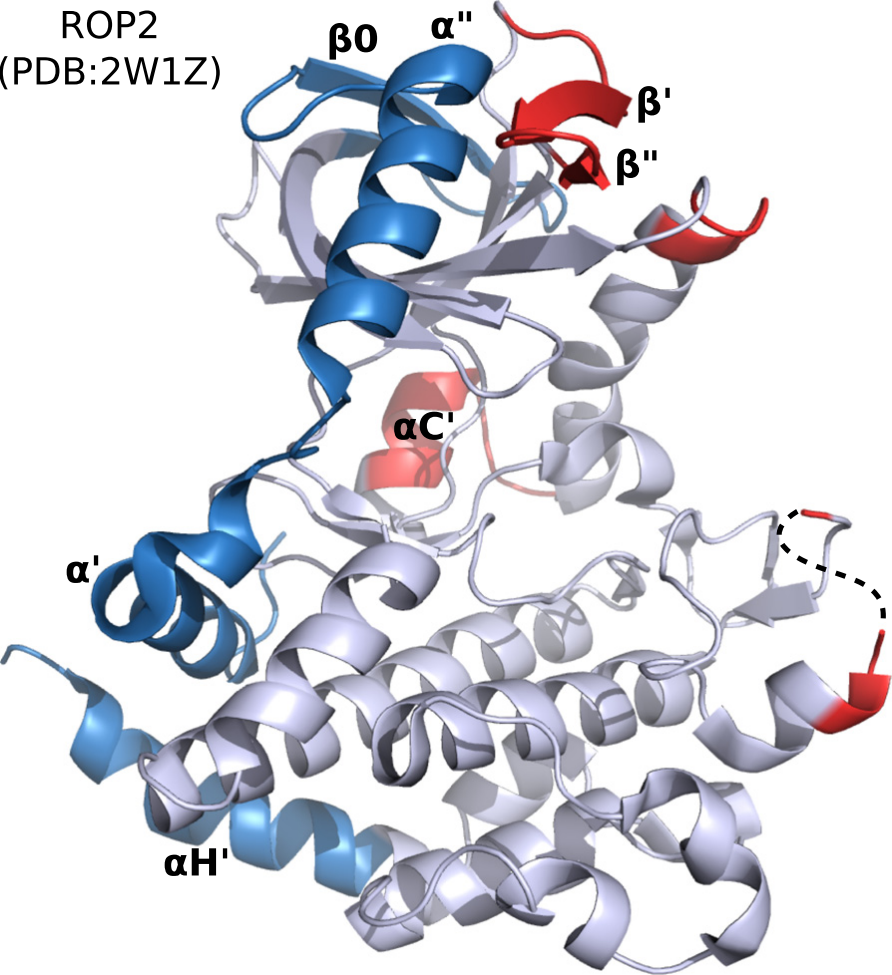
**Structural location of ROPK-conserved inserts.** Inserts relative to the conserved ePK fold are highlighted in red. The N-terminal extension (NTE) and C-terminal extended helix (*α*H’) are shown in blue. Novel secondary structures are labeled according to Labesse et al. [36].

ROPK- and subfamily-specific inserts within the kinase domain are widespread, suggesting unique functional adaptations [36,37,50]. We found six conserved inserts in the ROPK domain relative to the PK domain (Figure 5). They are: 

(i) An extension of the *β*3- *α*C loop, residues 289–293 ^ROP2^, of varying length across ROPK subfamilies; it is fairly short (4–5 amino acids) in the NTE-bearing clade, missing altogether in ROPKL, but extends up to 13 amino acids other ROPKs including the *E. tenella*-specific clade.

(ii) C-terminal to the *α*C helix, residues 309–318 ^ROP2^, present in all subfamilies except the ROPKL clade in roughly equal size. In the ROP2/8 structures [PDB:2W1Z,3DZO,3BYV] it was observed to form an additional helix, termed *α*C’ [36], in the kinase inter-lobe hinge area (discussed below), while in the ROP5 structures [PDB:3Q5F,3Q60] it is disordered.

(iii) In *β*4– *β*5 loop, residues 335–351 ^ROP2^, present in most subfamilies, including ROP33 but not the other ROPKLs, in similar size. In a ROP2 structure [PDB:2W1Z] this appears as two *β* strands, termed *β*’ and *β*”, that extend the loop to form a *β*-hairpin in the kinase N-lobe [36], spatially near the *α*” helix of the NTE. In the other structure of ROP2, ROP8 and ROP5 [PDB:3DZO,3BYV,3Q5F,3Q60] this region is mostly disordered, though the protein sequences indicate the insert is present in this subfamily as well.

(iv) Between the kinase APE motif (end of the activation segment) and the *α*F helix, residues 453–462 ^ROP2^, present in varying lengths across the ROPK subfamilies including each of the major clades (NTE, Eten, ROPKL). This is near the substrate-binding site in typical protein kinases. The insert appears as a short 4aa loop in ROP5 [PDB:3Q60], but in ROP2 [PDB:3DZO] and ROP8 [PDB:3BYV] it forms an additional single-turn helix in crystal structures [PDB:3DZO, PDB:3BYV] [50], though this feature may have been stabilized in the crystals because of crystal packing.

(v) An extension of the *αF*– *α*G loop, absent from ROP2/8, ROP40 and ROP49 and the ROPKL clade, but present in ROP5 and the other ROPK subfamilies in the region of residues 467–478 ^ROP5^. In the ROP5 structures [PDB:3Q5F,3Q60], B-factors indicate this elongation of the *αF*– *α*G loop is relatively flexible compared to the adjacent regions; the G-helix itself appears unfolded. Sequences of other ROPKs, including ROP24, suggest it is even longer in those subfamilies.

(vi) In the *α*G– *α*H loop, near the C-terminus of the *α*G helix, a 5aa insert absent from ROP2/8, ROP5, ROP18, ROP23, ROP25, ROP26, ROP30 and ROP40 and the ROPKLs but present in the other ROPK subfamilies including the *E. tenella*-specific clade. The ROPKLs appear to have large deletions in this region, and may be missing the *α*G helix structure altogether. We note that the *α*G– *α*H loop is extended in many other protein kinases, most notably CMGC kinases [54].

### Distinguishing ROPK-specific conserved sites in the protein kinase domain, and corresponding structural features

We evaluated shifts in site-specific residue conservation between the ROPK family and overall PK superfamily by performing a goodness-of-fit test of residue frequences in the two sequence sets at each aligned column of the PK domain (see Methods). The same comparisons were also performed with each subfamily versus the other ROPKs (Additional file 2).

#### Hinge region

**Figure 6:**
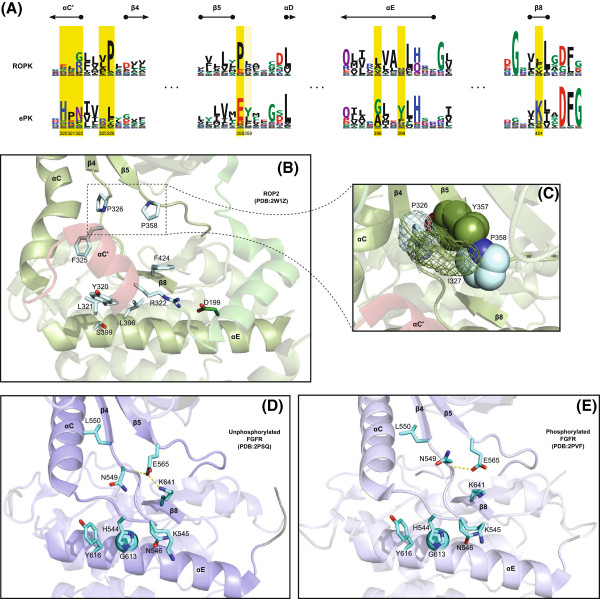
**Contrasting sites between ROPK and PK: kinase hinge region.(A)** Sequence logos of regions surrounding the *α*C’ helix insert and kinase hinge in ROPK (top) and PK (bottom), with selected contrasting sites highlighted. **(B)** ROP2 structure with selected contrasting sites shown in “sticks” representation. **(C)** Inset of the ROP2 structure around the contrasting prolines in the *α*C’- *β*4 loop and linker. Two other adjacent residues, I327 and Y357, pack against the prolines on opposite sides. **(D)** and **(E)** Two structures of the protein kinase FGFR show the conditional salt bridge between the linker glutamate (E565) and *β*8 lysine (K641), commonly observed in typical protein kinases, dependent upon kinase activation.

The most statistically significant sites distinguishing ROPKs from PKs overall are in the kinase hinge region. Numbered according to ROP2 [PDB:2W1Z], these are: sites [E/R/Y]320, L321, [R/G]322, [V/L/A]325 and P326 in the *α*C’– *β*4 loop; P358 in the *β*5– *α*D loop, and [L/F/Y]424 in the *β*8 strand (Figure 6). Two residues in the *α*E helix, [L/A/S]396 and [H/N/S]399, are oriented toward the hinge region and under the *α*C’ helix.

The residue P358 ^ROP2^ is typically a glutamate in most eukaryotic protein kinases (e.g. E121^PKA^, E565^FGFR^), where it contributes to the opening/closing motion of the kinase during activation by forming a lobe-bridging salt bridge interaction [55]. In fibroblast growth factor receptor kinase (FGFR), for example, the equivalent residue E565 hydrogen-bonds with K641 in the *β*8 strand conditionally upon phosphorylation of the FGFR activation loop [56] (Figure 6D,E). In ROP2, the residues equivalent to E565 and K641 are P358 and F424, respectively (Figure 6A,B). Since proline and phenylalanine are not charged residues, the ROP2 structure is incapable of forming the same interaction. The residue P358 ^ROP2^ is conserved as a proline throughout most of the ROPK family, with the exception of subfamilies ROP18 (methionine), ROP21/27 (aspartate, though a Phe appears in the *β*8 strand), ROP26 (serine), ROP32 (histidine), ROP41 (lysine), and the *E. tenella*-specific subfamilies (retained as glutamate, though only ROPK-Eten1 also retains a basic residue in the *β*8 strand) (Additional file 2).

The residues at sites P358 ^ROP2^ and P326 ^ROP2^ appear to have instead taken on another structural role. In ROPKs, the residue immediately N-terminal to P358 ^ROP2^, a site known as the kinase “gatekeeper” residue, is a large, usually hydrophobic residue oriented toward the *α*C’- *β*4 strand and, in the ROP2 structure, packing against the ROPK-conserved P326; the hydrophobic residue immediately N-terminal to P326 (most commonly valine but also varyingly leucine, alanine, phenylalanine, isoleucine and methionine in ROPKs) is likewise oriented toward the linker in the ROP2 structure, packing against P358 (Figure 6C). These four residues thus form a stable packing “box” bridging the *α*C’- *β*4 and *β*5- *α*D loops.

#### F-helix “WC” motif and disulfide bridge

**Figure 7:**
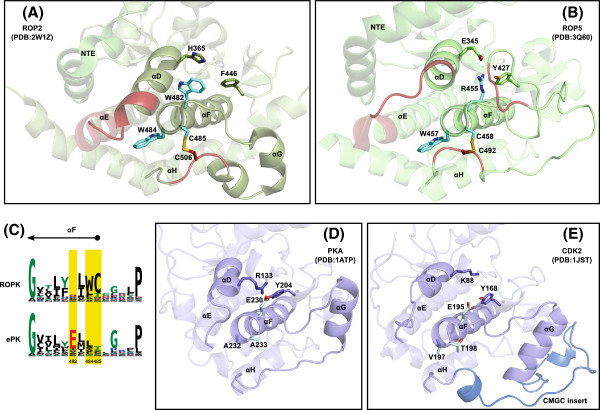
**Contrasting sites between ROPK and PK: C-lobe WC motif and loss of Glu constraint.(A)** and **(B)** Structures of ROP2 and ROP5 with WC motif and ROPK-conserved disulfide bridge residues shown in “sticks” representation. **(C)** Sequence logos of F helix region in ROPK (top) and ePK (bottom), with contrasting sites highlighted. **(D)** PKA, a representative typical protein kinase, with equivalent residues shown as sticks. **(E)** CDK2, another typical protein kinase. The “CMGC insert” occurs in the *αG*–*α*H loop but does not perform the same structural role as the ROPK-specific insert in the same region.

A distinctive “WC” motif appears at the end of the *α*F helix (Figure 7) in most ROPKs. The cysteine (C485 ^ROP2^), together with another ROPK-conserved cysteine (C506 ^ROP2^) [9] in the *α*G– *α*H insert described above, forms a disulfide bond which has been proposed to stabilize the two helices [50]. The tryptophan (W484 ^ROP2^) appears to pack against the extended *α*D and *α*E helices, pushing the *α*E helix futher outward. Thus the “WC” motif couples two ROPK-specific inserts to the substrate-binding lobe of the kinase core. There are no other known protein kinase families or subfamilies in which cysteines at the end of the F-helix and in the *αG*– *α*H loop co-occur in positions that could potentially interact. Additionally, both the WC motif and the *α*G– *α*H cysteine are absent from the *E. tenella* and ROPKL clades.

Another site in the *α*F helix (W482 ^ROP2^) is conserved as a glutamate in most ePKs (E230 ^PKA^), but unconserved in ROPKs, suggesting that a selective constraint that conserves glutamate at this site in most ePKs has been lost in the ROPK family. In at least some other ePKs, it appears that this glutamate can interact with a basic residue on the polar/charged surface of the amphipathic *α*D helix (R133^PKA^), as well as a conserved tyrosine in the P+1 pocket (Y204^PKA^) at the end of the activation segment (Figure 7D,E). Notably, the mutation of E230 to glutamine in PKA not only disrupted substrate recognition and phosphoryl transfer, but also resulted in higher temperature factors in the *α*D helix, particularly in R133 [57]. However, in ROPKs the interaction between the F and D helices occurs somewhat differently: in ROP5, R455 interacts with E345 and Y427, and in ROP2, W482 packs with H365, while the P+1-pocket Tyr replaced by F446, a side chain not capable of hydrogen bonding (Figure 7A,B).

### N-terminal extension to the protein kinase domain

Structural studies of ROP2, ROP8 and ROP5 revealed another feature common to each of these proteins, an N-terminal extension (NTE) to the canonical protein kinase domain consisting of at least two additional helices and a beta sheet, with the region between the two helices varying between ROP2/8 and ROP5 [36,37,50]. The NTE has also been suggested to be present in ROP18, ROP4/7 and ROP17 based on sequence homology, though its presence does not appear to be universal among rhoptry kinases [37,50]. We investigated the distinguishing features of NTE-containing rhoptry kinases to determine whether other ROPKs may also contain the NTE, and to look for additional conserved features that characterize this gene clade (see Methods).

**Figure 8:**
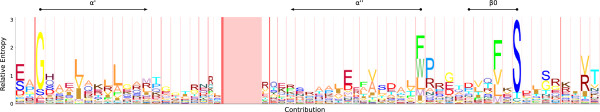
**HMM sequence logo of the NTE region.** Conserved secondary structures are indicated above the corresponding sequence positions. Generated with the HMM-Logos server LogoMat-M [58].

In addition to ROP2/8 and ROP5, we found significant matches in ROP4/7, ROP17 and ROP18, as expected, and also a number of additional subfamilies which appear to form a clade (Figure 1): ROP23, ROP24 (originally known as ROP2L8 [6]), ROP31, ROP40, ROP42/43/44, and the proposed ROP47. Four proteins in the ROPK-Unique (species-specific) category also showed evidence for NTE homology: TGME49_296000 (TGME49_096000 in ToxoDB prior to version 8.0), also known as ROP2L12 and previously identified as a pseudogene [6]; its orthologs TGVEG_050080 and TGGT1_054010; and the *E. tenella* protein ETH_00005190. A small number of sites in the NTE sequence region show strong conservation (Figure 8).

Having identified the NTE-bearing clade, we then compared this clade to all other identifid ROPKs to identify clade-specific residue conservation patterns. In the solved structures of ROP2, ROP8 and ROP5, several of these distinctive sites in the NTE clade are spatially located around the NTE itself, primarily near the conserved *β*0 and *α*’ secondary structure elements. In ROP2, V330 and P333 in the *β*4 sheet *β*4- *β*4’ loop are positioned on either side of the *β*0 sheet of the NTE, close to the conserved S244; in ROP5, the equivalent residues are V310 and Q313. In each of the solved crystal structures of ROP2 [PDB:2W1Z], ROP8 [PDB:3BYV] and ROP5 [PDB:3Q60], the *β*0 sheet passes directly between these two side chains, suggesting a structural selective constraint in NTE-bearing ROPKs.

Three significantly contrasting sites in the E-helix may also have some bearing on the NTE conformation or placement: H378 near the *α*E N-terminus, oriented toward the NTE in the ROP2 structure [PDB:2W1Z]; V382, a small, nonpolar residue oriented toward the extended *α*D; and Q388 in the middle of the *α*E helix, where in the ROP2 structure it interacts with the backbone of the conserved G198 at the N-terminus of the NTE *α*’ — though in the ROP5 structure the equivalent residue is I368 which despite having the same orientation cannot form an identical interaction.

Also in the *α*E helix, a hydrophobic residue (L391^ROP2^, A371^ROP5^), in place of a usually basic residue outside the NTE clade, is oriented toward a helix which extends beyond the kinase C-terminus in the ROP2, ROP8 and ROP5 structures, previously described as the *α*H’ helix [36]. Though this short, weakly conserved region is difficult to detect by sequence analysis, the conservation of the hydrophobic residue in the *α*E helix and the presence of this helix in the available structures does suggest a correlation between the presence of the NTE and C-terminal *α*H’ helix.

## Discussion

We classified the ROPKs into likely active kinases, likely pseudokinases, and predicted kinases that may be active, but with a noncanonical catalytic mechanism, based on differences in ePK-conserved residues surrounding the ATP binding pocket. Our alignment shows that conserved residues in or near the key ePK-conserved motifs, including the histidine of the canonical “HRD” motifs, are well aligned for each of these categories, so it is unlikely that the absence of the key aspartates in predicted pseudokinases is due to misalignment. Structural investigation of the unusual motifs in noncanonical subfamilies ROP24 and ROP45 in *T. gondii* could reveal novel kinase mechanisms of activation, ATP positioning and catalysis. Relatedly, analysis of the equivalent motifs in the ROPK pseudokinases could improve our understanding of pseudokinases in general.

Our phylogenetic tree of ROPK subfamilies revealed three specific clades of interest: the NTE-bearing ROPKs, the only clade for which crystal structures have been solved or even homology models reliably constructed; an *E. tenella*-specific expansion of ROPKs; and the divergent, intron-bearing ROPKLs. Notably, each of these clades contains both predicted active kinases and pseudokinases, indicating a pattern of evolution in which, in a parsimonious interpretation, pseudokinases repeatedly emerge from an ancestral state shared with active kinases, rather than a single or small number of expansions of pseudokinases.

We were unable to find conclusive published evidence that the ROPKL proteins are indeed localized to the rhoptry during the tachyzoite stage of coccidians and expelled during invasion at the same time and through the same mechanism as other ROPKs. ROP35 protein expression has been detected during the *T. gondii* tachyzoite stage [59] and the *E. tenella* merozoite stage (ETH_00005905) [38]. Signal peptides were predicted for ROP33, ROP50 and BPK1, but not ROP35, while the gene models of ROP34 and ROP46 contain a short or nonexistent N-tail to the kinase domain which could indicate a trunctated gene model. However, transcription levels across the cell cycle do not match the distinctive two-peaked pattern of *T. gondii* rhoptry proteins in any of the *T. gondii* ROPKLs [60]; the secretory organelle of BPK1 was not identified in the study that described the protein [44]. Our HMM profile search and gene trees indicated that the ROPKL proteins show stronger sequence similarity to typical ROPKs than to any other characterized protein kinase family, leaving open the question of how deep their functional similarity goes.

A common theme we observe in structural features unique to the ROPK family is the interaction between ROPK-specific inserts or structural motifs, including the N-terminal extension (NTE), and conserved sites within the kinase domain that show contrasting selection in ROPKs. Two regions in particular, the kinase hinge region surrounding the *α*C’ helix and and the dusulphide bridge at the end of the *α*F helix, suggest several possible functional or mechanistic consequences.

Our observations in the ROPK hinge region raise several hypotheses. The *α*C’ insert in the *αC*– *β*4 loop has possible structural analogues in other kinases. The vaccinia-related kinase (VRK) family has a similar insert which packs hydrophobically against the *α*E helix and was proposed to promote a closed conformation of the kinase domain in the pseudokinase VRK3 [61]; the authors of that study suggested that related active kinases that retain the same feature would be constitutively active. Comparison of the structure of VRK3 [PDB:2JII] to that of ROP2 [PDB:2WIZ] indicates that the ROPK-conserved site L396^ROP2^ (Figure 6A,B) may perform a similar role to the VRK3-conserved F296^VRK3^ in hydrophobically coupling the two lobes of the kinase domain. Interestingly, the ATP-bound and *apo* structures of the pseudokinase ROP5 show very little overall conformational change [37]. As another example, crystal structures of the yeast SRPK protein Sky1 conserve a short *α*C’ helix insert, and the flexibility of this region is indicated to be critical for interlobe closure [62]. Together with the ROPK-specific conservation of prolines in the *αC*– *β*4 loop and linker, this could indicate the possibility that these differences modulate interlobe closure (the kinase hinging mechanism) in ROPKs.

Another hypothesis regarding the function of the *α*C’ helix, not necessarily conflicting with the above hypothesis, is that it could serve as a binding interface or protein-protein interaction site. We observed that the *α*C’ helix does not pack hydrophobically against the N-lobe of the kinase domain in the available ROP2 structures; instead, there appear to be water molecules in between [PDB: 3Q60] [37]. The B-factors are somewhat higher than in the immediately surrounding areas, and the symmetry of the ROP2 structure suggests that the insert may have been stabilized in this structure by crystal packing. Given that the same region is disordered in the available ROP5 structures, it appears possible that *α*C’ may be relatively flexible, capable of unfolding from the helical secondary structure into a mobile loop. For comparison, in VRK3, a surface patch centered on the *αC*– *α*C’ region has been proposed as a binding site [61].

In the kinase C-lobe, a pair of ROPK-conserved cysteines form a disulfide bridge between the end of the *α*F helix and the *αG*– *α*H loop, which is extended in most ROPKs. A conserved tryptophan adjacent to the *α*F cysteine packs hydrophobically against the *α*D and *α*E helices, which are also extended in ROPKs; thus the “WC” motif appears to couple both ROPK inserts to the kinase C-lobe. Notably, this stabilization occurs in the surface region of the protein that was identified as polymorphic between ROP5 alleles in *T. gondii*[37], and was recently shown to be the interface of an interaction with the host (mouse) immunity-related GTPase (IRG) protein [19]. Reese et al. proposed an allosteric network involving the NTE and *α*F helix to link the polymorphic surfaces in the C-lobe and kinase active site in ROP5 [37]. The variability of this site in ROPKs may therefore be justified by its involvement in that network, which itself appears to be variable in ROPKs. We can hypothesize that, at least in ROP5, the increased structural stability provided by the WC motif in this region permits these subfamily-specific mutations to proliferate at this surface without compromising the folding or stability of the kinase C-lobe [63]. This hypothesis assumes that the disulfide bridge is indeed maintained throughout the lifespan of the protein; while it appears as such in the available solved structures, we note that once the protein is inside the host cell, the cytosolic environment is not conducive to disulfide bond formation. The two cysteines involved are co-conserved in not only the PVM-associated ROP2, ROP8, ROP5 and ROP18, but also ROP16, which has been shown to be localized to the host nucleus [22], among other ROPKs.

We also searched for sites that showed conservation specific to the NTE-bearing ROPK clade, rather than ROPKs as a whole. Interestingly, only a small number of strongly contrasting sites emerged as specific to this clade. This could indicate that the mechanistic roles of the NTE vary across even the NTE-bearing clade of ROPKs.

More structural information will be essential to further understand the ROPK family. Currently, only ROPKs from the ROP2/8 and ROP5 subfamilies within the NTE clade have been solved [36,37,50]. While these structures have been invaluable in understanding ROPK mechanisms and possible functions, the low sequence identity and presence of indels across subfamilies makes it difficult to produce reliable homology models for ROPK subfamilies outside this clade. We can suggest several important ROPKs outside the NTE clade which appear to be active kinases, are highly expressed [10], and from which we could gain important insights from the solved crystal structure. ROP16 was indirectly implicated in virulence differences between *T. gondii* strains in mice [15], and also shown to to modulate the host STAT3 and STAT6 pathway response [22-26], but the precise mechanisms of this action remain to be discovered. Peixoto *et al.*[10] found evidence that ROP38 is involved in modulating the MAPK cascade; the ROP19/29/38 subfamily was also found to be independently duplicated in *T. gondii* and *N. caninum*, thus the other subfamily members could easily be modeled if a ROP38 structure were available. Finally, ROP35 is a representative member of the divergent, poorly understood ROPKL clade; the presence of several indels relative to other ROPKs at structurally important locations in the sequence suggest that a crystal structure would almost certainly reveal surprising variations on the ePK fold and catalytic mechanisms.

## Conclusion

In this study, we developed novel bioinformatic methods to study patterns of diversification and neofunctionalization in the rhoptry kinase family, and integrated the results of a systematic, multi-species analysis with the structural context provided by the solved structures. Our phylogenetic analysis revealed a subfamily-level structure shared across species, as well as lineage-specific expansions within the ROPK family and three distinct sub-clades of ROPK. We applied general knowledge of protein kinase mechanisms to categorize each rhoptry kinase as a likely active, likely pseudokinase, or potentially active but with an atypical catalytic mechanism. We determined the sequence and structural features that distinguish these subfamilies from each other, as well as those that distinguish the ROPK family as a whole from typical ePKs. Where possible, ROPK-specific motifs were placed into structural context to develop functional hypotheses.

This work sheds light on several important but previously unrecognized features shared among rhoptry kinases, as well as the essential differences between active and degenerate protein kinases or pseudokinases. Our studies provide specific hypothesis for further characterizing ROPK structure and function and also inform ongoing efforts to design protein kinase inhibitors for global diseases caused by coccidian parasites.

## Methods

### Data collection

The sequences of translated gene models, unannotated genomes and ESTs from the species *Toxoplasma gondii*, *Neospora caninum*, *Eimeria tenella* were retrieved from ToxoDB version 8.1 [64]. Pre-release genomic sequences and ESTs of *Sarcocystis neurona* were provided by the laboratories of Dan Howe, Christopher Schardl and Jessica Kissinger.

After constructing the initial ROPK subfamily profiles (below), additional ROPK sequences were identified in the NCBI databases est_others and nr and added to the profiles. To obtain putative ROPK sequences from the unannotated *T. gondii* and *S. neurona* genomes, we used the program exonerate (https://www.ebi.ac.uk/~guy/exonerate/; also see [65]) to align the ROPK subfamily consensus sequences to each genome scaffold sequence, omitting introns according to likely splice sites. A script using Biopython [66] was then used to extract the highest-scoring putative protein sequences from the exonerate output and combine identical sequences and sequence fragments.

### Subfamily classification

We previously constructed a database of HMM profiles for every known protein kinase family and subfamily defined in KinBase [67], as well as several apicomplexan-specific kinase families [11]. The ROPK profile in this set was initially constructed from annotated ROPK sequences in ToxoDB, similar to the techinique described by Peixoto *et al.*[10]. Sequences were aligned using MAFFT version 6.940 [68] with a “seed” alignment of the protein kinase domain constructed using published PDB structures [PDB: 2W1Z, 3BYV, 3DZO, 3Q5Z, 3Q60] [36,37,50] and the structure alignment program TM-align (May 2012 release) [69]. Finally, HMM profiles were constructed from each sequence alignment and compiled into an HMM profile database (Additional file 3). We used this HMM profile database to search the protein and translated EST sequences described in the previous section; those which scored as stronger matches to the ROPK-specific HMM profile than to our ePK profiles were taken as an initial set of putative rhoptry kinases.

We developed a program called Fammer to partially automate the construction and curation of hierarchical protein subfamily sequence profiles for use with HMMer 3.0 [70] and MAPGAPS 1.0 [71], and to use these HMM and MAPGAPS profiles for sequence search, classification and alignment. The Fammer software package, including source code, documentation and the ROPK sequence profiles used in this study, is available at http://github.com/etal/fammer.

The full-length ROPK sequences identified in each annotated coccidian genome and translated EST set were clustered using OrthoMCL version 2.0.3 [72]. We manually trimmed the sequences in each OrthoMCL cluster to the canonical protein kinase domain and aligned the sequence sets with Fammer version 0.1.0 to create an initial set of ROPK subfamily profiles, as well as a set of “unique” or orphan ROPKs which matched the ROPK HMM profile but were not placed into a larger cluster by OrthoMCL.

Iteratively, we performed the following steps to refine the ROPK subfamily classification. We constructed a phylogenetic tree of the consensus sequences of each putative ROPK subfamily, using FastTree version 2.1.5 [73], and merged ortholog groups which were separated by short branches in the tree and, for subfamilies that appeared in multiple copies within a single genome (e.g. ROP2/8, ROPK-Eten3), showed co-localization in the chromosome. Existing descriptions of the annotated *T. gondii* proteins were used to assign names to subfamilies. Unannotated subfamilies that were phylogenetically placed basally to the known ROPKs, indicating closer relationship to other ePKs, were removed. We visually inspected each subfamily sequence set for potential outlier sequences, on the basis of conserved motifs in key regions of the kinase domain, and moved any of these to the “unique” sequence set. We used the Fammer *build* command to realign all sequences and to construct an HMM profile database of all subfamily profiles, then used this database with the Fammer *scan* command to reclassify the “unique” or outlier ROPK sequences. We included a profile of non-ROPK protein kinase sequences in this HMM database in order to identify and remove false positives in the “unique” set as well as subsequent searches of the coccidian proteome, genome and EST sequences. Finally, we used the Fammer *refine* command to perform leave-one-out validation of each subfamily profile versus the “unique” sequence set, following the approach described by Hedlund *et al.*[74]. This process yielded 42 stable subfamilies of ROPK, along with a “ROPK-Unique” profile set of unclassified orphan sequences. We then identified the ROPK complement in each annotated proteome by running the Fammer *scan* command with the final ROPK HMM profile database, each coccidian species’ proteome sequences, and an expectation-value cutoff of 10^−10^.

### Subfamily tree inference

We used the curated alignment of consensus sequences from each ROPK subfamily profile and the non-ROPK protein kinase profile as input to infer phylogenic trees. To quickly examine the structure of the ROPK family during profile refinement, we used FastTree [73] with the WAG scoring matrix, gamma model of rate variation and pseudocount correction for gaps. To infer the final tree shown in Figure 1, we first used the GUIDANCE server [75] with 100 replicates of PRANK and removed columns with less than 5% support, in order to remove alignment columns that were likely to have been misaligned while retaining most of the potentially phylogenetically informative columns. We then used a script to remove columns that were more than 30% gap characters. This filtering yielded an alignment of 279 columns, slightly less than the length of the top-level ROPK HMM profile (288 columns). We inferred the tree from this alignment using PhyML (December 2011 release) [76], with the LG scoring matrix, gamma model of rate variation, empirically estimated amino acid frequencies and 100 bootstrap runs, taking the output of FastTree as the user-supplied starting tree. Finally, we used script based on the Bio.Phylo module of Biopython [77] to reroot the tree with ePK as the outgroup, collapse all splits with less than 25% bootstrap support, colorize the specific clades of interest and visualize the tree. The alignment of subfamily consensus sequences and the inferred tree have been deposited in TreeBase (http://www.treebase.org/; Study ID: 14212).

### Analysis of evolutionary constraints

To identify sites of contrasting conservation between ROPK subfamilies, and between all ROPKs and the broader protein kinase superfamily, we compared aligned sites between two given sequence sets by applying a multinomial log-likelihood test (G-test) [78] of the residue compositions of each column in the two sets. The test statistic *G* is derived from the frequencies of each amino acid type as observed in the “foreground” set, *O*_*i*_, and as expected based on the “background” set, *E*_*i*_, including pseudocounts taken from the amino acid frequencies of the full alignment.

$$G=2\underset{i\in \mathrm{a.a.}}{\sum }O_{i}\ln\frac{O_{i}}{E_{i}}$$

To adjust for the non-independence of sequences in each set due to phylogenetic relatedness, the aligned sequences in each set are weighted according to the Henikoff heuristic [79], and the amino acid counts in each column are adjusted according to these sequence weights, an approach also used in PSI-BLAST [80]. The test statistic *G* follows the chi-squared distribution with 19 degrees of freedom (for the 20 amino acid types).

We implemented this test in a program called CladeCompare, available at http://github.com/etal/cladecompare. The output of the program includes (i) a table of the probabilities (p-values) of each site in the combined alignment, (ii) a list of the significantly contrasting sites after adjusting for multiple testing using the Benjamini-Hochberg false discovery rate method [81], and (iii) images of paired “background” and “foreground” sequence logos to illustrate the contrast at significant sites, generated using the WebLogo [82] and ReportLab [83] libraries.

### Detection of the N-terminal extension in additional subfamilies

To identify which ROPK subfamilies share sequence homology to the NTE region observed in the ROP2, ROP8 and ROP5 structures, and suggested to be present in ROP18, ROP4/7 and ROP17, we used the CHAIN program [84] with the previously identified NTE-bearing sequences as the query set and the complete set of full-length ROPK sequences as the main set. CHAIN identified a “foreground” partition corresponding to the clade highlighted in Figure 1.

We then constructed an alignment of the sequence regions N-terminal to the kinase domain in the identified using the “accurate” mode of T-Coffee [85], built an HMM profile from this alignment, and used HMMer 3.0 [70] to search the full-length ROPK sequences. This recovered the same ROPK subfamilies identified by CHAIN, confirming the presence of homologous NTE regions in those subfamilies.

### Structural analysis

Sites of interest were mapped onto PDB protein structures with a script and visualized in PyMOL [86] for manual inspection.

## Availability of supporting data

The data sets supporting the results of this article are available in the TreeBase repository, http://purl.org/phylo/treebase/phylows/study/TB2:S14212.

## Competing interests

Both authors declare that they have no competing interests.

## Authors’ contributions

ET performed the bioinformatics analyses. ET and NK conceived and designed the study, examined sequences and structural features and wrote the manuscript. Both authors read and approved the final manuscript.

## Supplementary Material

Additional file 1 **ROPK subfamily members in coccidian genomes.** Classification of each rhoptry kinase gene in selected genomes into the identified subfamilies. Click here for file

Additional file 2 **Conservation contrasts in aligned ROPK subfamily consensus sequences.** Alignment of the ROPK family consensus and the consensus sequences of each subfamily. The first row in each block indicates contrasting features of the ROPK family sequence profile versus the protein kinase superfamily, while all other rows indicate patterns specific to each subfamily profile. Profile-specific inserts are shown in yellow, relative deletions in gray. The statistical significance of the contrast in residue composition at each site is shown as a heat map, with non-significant sites in shades of blue, p-values between 0.05 and 0.01 in white, and p-values less than 0.01 in increasingly dark shades of red. Significant sites in the ROPK-PK comparison are indicated with asterisks along the top of the alignment. This visualization was generated by CladeCompare version 0.1.0. Click here for file

Additional file 3 **ROPK subfamily HMM profile database.** HMMer 3.0 database containing profiles for each individual ROPK subfamily, the ROPK family as a whole, and typical protein-kinase-like (PKL) sequences which can be used to distinguish ROPKs from other protein kinases. Click here for file

## References

[B1] Montoya JG Liesenfeld O Toxoplasmosis Lancet 2004 363 9425 1965 1976 15194258 10.1016/S0140-6736(04)16412-X

[B2] Kim K Weiss L M Toxoplasma: the next 100 years Microbes Infect 2008 10 9 978 984 10.1016/j.micinf.2008.07.015 PMC2596634 18672085

[B3] Kim K Weiss L M Toxoplasma gondii: the model apicomplexan Int J Parasitol 2004 34 3 423 432 15003501 10.1016/j.ijpara.2003.12.009 PMC3086386

[B4] Sibley L D Invasion and intracellular survival by protozoan parasites Immunol Rev 2011 240 72 91 21349087 10.1111/j.1600-065X.2010.00990.x PMC3697736

[B5] Morrissette NS Sibley LD Cytoskeleton of Apicomplexan Parasites Microbiol Mol Biol Rev 2002 66 21 38 11875126 10.1128/MMBR.66.1.21-38.2002 PMC120781

[B6] Boothroyd JC Dubremetz JF Kiss and spit: the dual roles of Toxoplasma rhoptries Nat Rev Microbiol 2008 6 79 88 18059289 10.1038/nrmicro1800

[B7] Hunter Ca Sibley LD Modulation of innate immunity by Toxoplasma gondii virulence effectors Nat Rev Microbiol 2012 10 11 766 778 23070557 10.1038/nrmicro2858 PMC3689224

[B8] Bradley PJ Ward C Cheng SJ Alexander DL Coller S Coombs GH Dunn JD Ferguson DJ Sanderson SJ Wastling JM Boothroyd JC Proteomic analysis of rhoptry organelles reveals many novel constituents for host-parasite interactions in Toxoplasma gondii J Biol Chem 2005 280 40 34245 34258 16002398 10.1074/jbc.M504158200

[B9] Labesse G Dubremetz JF El Hajj H The ROP2 family of Toxoplasma gondii rhoptry proteins: proteomic and genomic characterization and molecular modeling Proteomics 2006 6 21 5773 5784 17022100 10.1002/pmic.200600187

[B10] Peixoto L Chen F Harb OS Davis PH Beiting DP Brownback CS Ouloguem D Roos DS Integrative genomic approaches highlight a family of parasite-specific kinases that regulate host responses Cell Host Microbe 2010 8 2 208 218 20709297 10.1016/j.chom.2010.07.004 PMC2963626

[B11] Talevich E Mirza A Kannan N Structural and evolutionary divergence of eukaryotic protein kinases in Apicomplexa BMC Evol Biol 2011 11 321 22047078 10.1186/1471-2148-11-321 PMC3239843

[B12] Miranda-Saavedra D Gabaldón T Barton GJ Langsley G Doerig C The kinomes of apicomplexan parasites Microbes Infect 2012 14 10 796 810 22587893 10.1016/j.micinf.2012.04.007

[B13] Lim DC Cooke BM Doerig C Saeij JPJ Toxoplasma and Plasmodium protein kinases: roles in invasion and host cell remodelling Int J Parasitol 2012 42 21 32 22154850 10.1016/j.ijpara.2011.11.007 PMC3428259

[B14] Bradley PJ Sibley LD Rhoptries: an arsenal of secreted virulence factors Curr Opin Microbiol 2007 10 6 582 587 17997128 10.1016/j.mib.2007.09.013 PMC2682365

[B15] Saeij JPJ Boyle JP Coller S Taylor S Sibley LD Brooke-Powell ET Ajioka JW Boothroyd JC Polymorphic secreted kinases are key virulence factors in toxoplasmosis Science 2006 314 5806 1780 1783 17170306 10.1126/science.1133690 PMC2646183

[B16] Taylor S Barragan A Su C Fux B Fentress SJ Tang K Beatty WL Hajj HE Jerome M Behnke MS White M Wootton JC Sibley LD A secreted serine-threonine kinase determines virulence in the eukaryotic pathogen Toxoplasma gondii Science 2006 314 5806 1776 1780 17170305 10.1126/science.1133643

[B17] Behnke MS Khan A Wootton JC Dubey JP Tang K Sibley LD Virulence differences in Toxoplasma mediated by amplification of a family of polymorphic pseudokinases Proc Natl Acad Sci U S A 2011 108 23 9631 9636 21586633 10.1073/pnas.1015338108 PMC3111276

[B18] Fentress SJ Mashayekhi M Li LX Taylor Ga Sibley LD Behnke M S The polymorphic pseudokinase ROP5 controls virulence in Toxoplasma gondii by regulating the active kinase ROP18 PLoS Pathogens 2012 8 11 e1002992 23144612 10.1371/journal.ppat.1002992 PMC3493473

[B19] Fleckenstein MC Reese ML Könen-Waisman S Boothroyd JC Howard JC Steinfeldt T A Toxoplasma gondii pseudokinase inhibits host IRG resistance proteins PLoS Biol 2012 10 7 e1001358 22802726 10.1371/journal.pbio.1001358 PMC3393671

[B20] Reese ML Zeiner GM Saeij JPJ Boothroyd JC Boyle JP Polymorphic family of injected pseudokinases is paramount in Toxoplasma virulence Proc Natl Acad Sci U S A 2011 108 23 9625 9630 21436047 10.1073/pnas.1015980108 PMC3111280

[B21] Steinfeldt T Könen-Waisman S Tong L Pawlowski N Lamkemeyer T Sibley LD Hunn JP Howard JC Phosphorylation of mouse immunity-related GTPase (IRG) resistance proteins is an evasion strategy for virulent Toxoplasma gondii PLoS Biol 2010 8 12 e1000576 21203588 10.1371/journal.pbio.1000576 PMC3006384

[B22] Saeij JPJ Coller S Boyle JP Jerome ME White MW Boothroyd JC Toxoplasma co-opts host gene expression by injection of a polymorphic kinase homologue Nature 2007 445 7125 324 327 17183270 10.1038/nature05395 PMC2637441

[B23] Yamamoto M Standley DM Takashima S Saiga H Okuyama M Kayama H Kubo E Ito H Takaura M Matsuda T Soldati-Favre D Takeda K A single polymorphic amino acid on Toxoplasma gondii kinase ROP16 determines the direct and strain-specific activation of Stat3 J Exp Med 2009 206 12 2747 2760 19901082 10.1084/jem.20091703 PMC2806617

[B24] Ong YC Reese ML Boothroyd JC Toxoplasma rhoptry protein 16 (ROP16) subverts host function by direct tyrosine phosphorylation of STAT6 J Biol Chem 2010 285 37 28731 28740 20624917 10.1074/jbc.M110.112359 PMC2937901

[B25] Ong YC Boyle JP Boothroyd JC Strain-dependent host transcriptional responses to Toxoplasma infection are largely conserved in mammalian and avian hosts PLoS ONE 2011 6 10 e26369 22022607 10.1371/journal.pone.0026369 PMC3192797

[B26] Butcher Ba Fox Ba Rommereim LM Kim SG Maurer KJ Yarovinsky F Herbert DR Bzik DJ Denkers EY Toxoplasma gondii rhoptry kinase ROP16 activates STAT3 and STAT6 resulting in cytokine inhibition and arginase-1-dependent growth control PLoS Pathog 2011 7 9 e1002236 21931552 10.1371/journal.ppat.1002236 PMC3169547

[B27] Doerig C Protein kinases as targets for anti-parasitic chemotherapy Biochim Biophys Acta 2004 1697 1-2 155 168 15023358 10.1016/j.bbapap.2003.11.021

[B28] Rotella DP Recent results in protein kinase inhibition for tropical diseases Bioorg Med Chem Lett 2012 22 22 6788 6793 23063403 10.1016/j.bmcl.2012.09.044

[B29] Knighton D Zheng J Ten Eyck L Ashford V Xuong N Taylor S Sowadski J Crystal structure of the catalytic subunit of cyclic adenosine monophosphate-dependent protein kinase Science 1991 253 5018 407 414 1862342 10.1126/science.1862342

[B30] Zeqiraj E Filippi BM Deak M Alessi DR van Aalten DMF Structure of the LKB1-STRAD-MO25 complex reveals an allosteric mechanism of kinase activation Science 2009 326 5960 1707 1711 19892943 10.1126/science.1178377 PMC3518268

[B31] Shi F Telesco SE Liu Y Radhakrishnan R Lemmon Ma ErbB3/HER3 intracellular domain is competent to bind ATP and catalyze autophosphorylation Proc Natl Acad Sci U S A 2010 107 17 7692 7697 20351256 10.1073/pnas.1002753107 PMC2867849

[B32] Boudeau J Miranda-Saavedra D Barton GJ Alessi DR Emerging roles of pseudokinases Trends Cell Biol 2006 16 9 443 452 16879967 10.1016/j.tcb.2006.07.003

[B33] Kornev AP Taylor SS Pseudokinases: functional insights gleaned from structure Structure 2009 17 5 7 19141276 10.1016/j.str.2008.12.005 PMC6226308

[B34] Zeqiraj E Pseudokinases-remnants of evolution or key allosteric regulators? Curr Opin Struct Biol 2010 20 6 772 781 21074407 10.1016/j.sbi.2010.10.001 PMC3014569

[B35] Reese ML Boyle JP Virulence without catalysis: how can a pseudokinase affect host cell signaling? Trends Parasitol 2012 28 2 53 57 22257555 10.1016/j.pt.2011.12.004

[B36] Labesse G Gelin M Bessin Y Lebrun M Papoin J Cerdan R Arold ST Dubremetz JF ROP2 from Toxoplasma gondii: a virulence factor with a protein-kinase fold and no enzymatic activity Structure 2009 17 139 146 19141290 10.1016/j.str.2008.11.005

[B37] Reese ML Boothroyd JC A conserved noncanonical motif in the pseudoactive site of the ROP5 pseudokinase domain mediates its effect on Toxoplasma virulence J Biol Chem 2011 286 33 29366 29375 21708941 10.1074/jbc.M111.253435 PMC3190742

[B38] Oakes RD Kurian D Bromley E Ward C Lal K Blake DP Reid AJ Pain A Sinden RE Wastling JM Tomley FM The rhoptry proteome of Eimeria tenella sporozoites Int J Parasitol 2013 43 2 181 188 23262303 10.1016/j.ijpara.2012.10.024

[B39] Lindsay DS Mitchell SM Vianna MC Dubey JP Sarcocystis neurona (Protozoa: Apicomplexa): description of oocysts, sporocysts, sporozoites, excystation, and early development J Parasitol 2004 90 3 461 465 15272465 10.1645/GE-230R

[B40] Dubey JP Lindsay DS Fritz D Speer CA Structure of Sarcocystis neurona sarcocysts J Parasitol 2001 87 6 1323 1327 11780816 10.1645/0022-3395(2001)087[1323:SOSNS]2.0.CO;2

[B41] Speer Ca Dubey JP Ultrastructure of schizonts and merozoites of Sarcocystis neurona Parasitol Vet 2001 95 2–4 263 271 10.1016/s0304-4017(00)00392-7 11223206

[B42] Petersen TN Brunak Sr von Heijne G Nielsen H SignalP 4.0: discriminating signal peptides from transmembrane regions Nat Methods 2011 8 10 785 786 21959131 10.1038/nmeth.1701

[B43] Morrison Da Bornstein S Thebo P Wernery U Kinne J Mattsson JG The current status of the small subunit rRNA phylogeny of the coccidia (Sporozoa) Int J Parasitol 2004 34 4 501 514 15013740 10.1016/j.ijpara.2003.11.006

[B44] Buchholz KR Fritz HM Chen X Durbin-Johnson B Rocke DM Ferguson DJ Conrad Pa Boothroyd JC Identification of tissue cyst wall components by transcriptome analysis of in vivo and in vitro Toxoplasma gondii bradyzoites Eukaryot Cell 2011 10 12 1637 1647 22021236 10.1128/EC.05182-11 PMC3232729

[B45] Taylor SS Knighton DR Zheng J Sowadski JM Gibbs CS Zoller MJ A template for the protein kinase family Trends Biochem Sci 1993 18 3 84 89 8480367 10.1016/0968-0004(93)80001-r

[B46] Scheeff ED Bourne PE Structural evolution of the protein kinase-like superfamily PLoS Comput Biol 2005 1 5 e49 16244704 10.1371/journal.pcbi.0010049 PMC1261164

[B47] Oruganty K Talathi NS Wood Za Kannan N Identification of a hidden strain switch provides clues to an ancient structural mechanism in protein kinases Proc Natl Acad Sci U S A 2013 110 3 924 929 23277537 10.1073/pnas.1207104110 PMC3549070

[B48] Xu B English JM Wilsbacher JL Stippec S Goldsmith EJ Cobb MH WNK1, a novel mammalian serine/threonine protein kinase lacking the catalytic lysine in subdomain II J Biol Chem 2000 275 22 16795 16801 10828064 10.1074/jbc.275.22.16795

[B49] Mukherjee K Sharma M Urlaub H Bourenkov GP Jahn R Südhof TC Wahl MC CASK Functions as a Mg2+-independent neurexin kinase Cell 2008 133 2 328 339 18423203 10.1016/j.cell.2008.02.036 PMC3640377

[B50] Qiu W Wernimont AK Tang K Taylor S Lunin V Schapira M Fentress S Hui R Sibley LD Novel structural and regulatory features of rhoptry secretory kinases in Toxoplasma gondii EMBO J 2009 28 7 969 979 19197235 10.1038/emboj.2009.24 PMC2670854

[B51] Eswaran J Lee WH Debreczeni JE Filippakopoulos P Turnbull A Fedorov O Deacon SW Peterson JR Knapp S Crystal Structures of the p21-activated kinases PAK4, PAK5, and PAK6 reveal catalytic domain plasticity of active group II PAKs Structure 2007 15 2 201 213 17292838 10.1016/j.str.2007.01.001 PMC1885963

[B52] Huse M Kuriyan J The conformational plasticity of protein kinases Cell 2002 109 3 275 282 12015977 10.1016/s0092-8674(02)00741-9

[B53] Kornev AP Haste NM Taylor SS Eyck LFT Surface comparison of active and inactive protein kinases identifies a conserved activation mechanism Proc Natl Acad Sci U S A 2006 103 47 17783 17788 17095602 10.1073/pnas.0607656103 PMC1693824

[B54] Kannan N Neuwald AF Evolutionary constraints associated with functional specificity of the CMGC protein kinases MAPK, CDK, GSK, SRPK, DYRK, and CK2alpha Protein Sci 2004 13 8 2059 2077 15273306 10.1110/ps.04637904 PMC2279817

[B55] Lamers MB Hubbard RE Williams DH Antson a a Structure of the protein tyrosine kinase domain of C-terminal Src kinase (CSK) in complex with staurosporine J Mol Biol 1999 285 2 713 725 9878439 10.1006/jmbi.1998.2369

[B56] Chen H Ma J Li W Eliseenkova AV Xu C Neubert Ta Miller WT Mohammadi M A molecular brake in the kinase hinge region regulates the activity of receptor tyrosine kinases Mol Cell 2007 27 5 717 730 17803937 10.1016/j.molcel.2007.06.028 PMC2094128

[B57] Wu J Yang J Kannan N Xuong Nh Ten Eyck LF Taylor SS Madhusudan Crystal structure of the E230Q mutant of cAMP-dependent protein kinase reveals an unexpected apoenzyme conformation and an extended N-terminal A helix Protein Sci 2005 14 11 2871 2879 16253959 10.1110/ps.051715205 PMC2253214

[B58] Schuster-Böckler B Schultz J Rahmann S HMM Logos for visualization of protein families BMC Bioinformatics 2004 5 7 14736340 10.1186/1471-2105-5-7 PMC341448

[B59] Treeck M Sanders JL Elias JE Boothroyd JC The phosphoproteomes of Plasmodium falciparum and Toxoplasma gondii reveal unusual adaptations within and beyond the parasites’ boundaries Cell Host Microbe 2011 10 4 410 419 22018241 10.1016/j.chom.2011.09.004 PMC3254672

[B60] Behnke MS Wootton JC Lehmann MM Radke JB Lucas O Nawas J Sibley LD White MW Coordinated progression through two subtranscriptomes underlies the tachyzoite cycle of Toxoplasma gondii PLoS ONE 2010 5 8 e12354 20865045 10.1371/journal.pone.0012354 PMC2928733

[B61] Scheeff ED Eswaran J Bunkoczi G Knapp S Manning G Structure of the pseudokinase VRK3 reveals a degraded catalytic site, a highly conserved kinase fold, and a putative regulatory binding site Structure 2009 17 128 138 19141289 10.1016/j.str.2008.10.018 PMC2639636

[B62] Nolen B Ngo J Chakrabarti S Vu D Adams Ja Ghosh G Nucleotide-induced conformational changes in the Saccharomyces cerevisiae SR protein kinase, Sky1p, revealed by X-ray crystallography Biochemistry 2003 42 32 9575 9585 12911299 10.1021/bi0344331

[B63] Bloom JD Labthavikul ST Otey CR Arnold FH Protein stability promotes evolvability Proc Natl Acad Sci U S A 2006 103 15 5869 5874 16581913 10.1073/pnas.0510098103 PMC1458665

[B64] Gajria B Bahl A Brestelli J Dommer J Fischer S Gao X Heiges M Iodice J Kissinger JC Mackey AJ Pinney DF Roos DS Stoeckert CJ Wang H Brunk BP ToxoDB: an integrated Toxoplasma gondii database resource Nucleic Acids Res 2008 36 Database issue D553—D556 18003657 10.1093/nar/gkm981 PMC2238934

[B65] Slater GSC Birney E Automated generation of heuristics for biological sequence comparison BMC Bioinformatics 2005 6 31 15713233 10.1186/1471-2105-6-31 PMC553969

[B66] Cock PJA Antao T Chang JT Chapman BA Cox CJ Dalke A Friedberg I Hamelryck T Kauff F Wilczynski B de Hoon MJL Biopython: freely available Python tools for computational molecular biology and bioinformatics Bioinformatics 2009 25 11 1422 1423 19304878 10.1093/bioinformatics/btp163 PMC2682512

[B67] Manning G Plowman GD Hunter T Sudarsanam S Evolution of protein kinase signaling from yeast to man Trends Biochem Sci 2002 27 10 514 520 12368087 10.1016/s0968-0004(02)02179-5

[B68] Katoh K Ki Kuma Toh H Miyata T MAFFT version 5: improvement in accuracy of multiple sequence alignment Nucleic Acids Res 2005 33 2 511 518 15661851 10.1093/nar/gki198 PMC548345

[B69] Zhang Y Skolnick J TM-align: a protein structure alignment algorithm based on the TM-score Nucleic Acids Res 2005 33 7 2302 2309 15849316 10.1093/nar/gki524 PMC1084323

[B70] Eddy SR Accelerated profile HMM searches PLoS Comput Biol 2011 7 10 e1002195 22039361 10.1371/journal.pcbi.1002195 PMC3197634

[B71] Neuwald AF Rapid detection, classification and accurate alignment of up to a million or more related protein sequences Bioinformatics 2009 25 15 1869 1875 19505947 10.1093/bioinformatics/btp342 PMC2732367

[B72] Li L Stoeckert CJ Roos DS OrthoMCL: identification of ortholog groups for eukaryotic genomes. Genome Res 2003 13 9 2178 2189 12952885 10.1101/gr.1224503 PMC403725

[B73] Price MN Dehal PS Arkin AP FastTree 2–approximately maximum-likelihood trees for large alignments PLoS ONE 2010 5 3 e9490 20224823 10.1371/journal.pone.0009490 PMC2835736

[B74] Hedlund J Jörnvall H Persson B Subdivision of the MDR superfamily of medium-chain dehydrogenases/reductases through iterative hidden Markov model refinement BMC Bioinformatics 2010 11 534 20979641 10.1186/1471-2105-11-534 PMC2976758

[B75] Penn O Privman E Ashkenazy H Landan G Graur D Pupko T GUIDANCE: a web server for assessing alignment confidence scores Nucleic Acids Res 2010 38 Web Server issue W23 W28 20497997 10.1093/nar/gkq443 PMC2896199

[B76] Guindon S Dufayard JF Lefort V Anisimova M Hordijk W Gascuel O New algorithms and methods to estimate maximum-likelihood phylogenies: assessing the performance of PhyML 3.0 Syst Biol 2010 59 3 307 321 http://www.atgc-montpellier.fr/phyml/20525638 10.1093/sysbio/syq010

[B77] Talevich E Invergo BM Cock PJA Chapman BA Bio.Phylo: a unified toolkit for processing, analyzing and visualizing phylogenetic trees in Biopython BMC Bioinformatics 2012 13 209 22909249 10.1186/1471-2105-13-209 PMC3468381

[B78] Dunning T Accurate methods for the statistics of surprise and coincidence Comput Linguist 1993 19 1 61 74

[B79] Henikoff S Henikoff JG Position-based sequence weights J Mol Biol 1994 243 4 574 578 7966282 10.1016/0022-2836(94)90032-9

[B80] Altschul SF Madden TL Schäffer aa Zhang J Lipman DJ Zhang Z Gapped BLAST and PSI-BLAST: a new generation of protein database search programs Nucleic Acids Res 1997 25 17 3389 3402 9254694 10.1093/nar/25.17.3389 PMC146917

[B81] Benjamini Y Hochberg Y Controlling the false discovery rate: a practical and powerful approach to multiple testing J R Stat Soc. Ser B (Methodological) 1995 57 289 300

[B82] Crooks GE Hon G Chandonia Jm Brenner SE WebLogo: a sequence logo generator Genome Res 2004 14 6 1188 1190 15173120 10.1101/gr.849004 PMC419797

[B83] The ReportLab PDF generation library Reportlab Inc 2010 http://www.reportlab.org/

[B84] Neuwald A F The CHAIN program: forging evolutionary links to underlying mechanisms Trends Biochem Sci 2007 32 11 487 493 17962021 10.1016/j.tibs.2007.08.009

[B85] Notredame C Higgins DG Heringa J T-Coffee: A novel method for fast and accurate multiple sequence alignment J Mol Biol 2000 302 205 217 10964570 10.1006/jmbi.2000.4042

[B86] Delano W The PyMOL Molecular Graphics System 2011 http://www.pymol.org/

