# Supplementary material for: Structural and evolutionary adaptation of rhoptry kinases and pseudokinases, a family of coccidian virulence factors
Source: BMC Evol Biol. 2013 Jun 6;13:117. doi: 10.1186/1471-2148-13-117 (PMC3682881; doi:10.1186/1471-2148-13-117)
Supplement: Additional file 2 — Conservation contrasts in aligned ROPK subfamily consensus sequences. Alignment of the ROPK family consensus and the consensus sequences of each subfamily. The first row in each block indicates contrasting features of the ROPK family sequence profile versus the protein kinase superfamily, while all other rows indicate patterns specific to each subfamily profile. Profile-specific inserts are shown in yellow, relative deletions in gray. The statistical significance of the contrast in residue composition at each site is shown as a heat map, with non-significant sites in shades of blue, p-values between 0.05 and 0.01 in white, and p-values less than 0.01 in increasingly dark shades of red. Significant sites in the ROPK-PK comparison are indicated with asterisks along the top of the alignment. This visualization was generated by CladeCompare version 0.1.0. [file 1471-2148-13-117-S2.html]

ROPK.gtest.noise, NTE.gtest.noise, ETEN.gtest.noise, ROPKL-v-ROPK.gtest.noise, BPK1.cma.noise, ROP11.cma.noise, ROP16.cma.noise, ROP17.cma.noise, ROP18.cma.noise, ROP19-29-38.cma.noise, ROP20.cma.noise, ROP21-27.cma.noise, ROP22.cma.noise, ROP23.cma.noise, ROP24.cma.noise, ROP25.cma.noise, ROP26.cma.noise, ROP2-8.cma.noise, ROP28.cma.noise, ROP30.cma.noise, ROP31.cma.noise, ROP32.cma.noise, ROP33.cma.noise, ROP34.cma.noise, ROP35.cma.noise, ROP36.cma.noise, ROP37.cma.noise, ROP39.cma.noise, ROP40.cma.noise, ROP41.cma.noise, ROP42-43-44.cma.noise, ROP45.cma.noise, ROP46.cma.noise, ROP4-7.cma.noise, ROP47.cma.noise, ROP48.cma.noise, ROP49.cma.noise, ROP50.cma.noise, ROP5.cma.noise, ROPK-Eten1.cma.noise, ROPK-Eten2a.cma.noise, ROPK-Eten2b.cma.noise, ROPK-Eten3.cma.noise, ROPK-Eten4.cma.noise, ROPK-Eten5.cma.noise, ROPK-Eten6.cma.noise, ROPK-Unique.cma.noise


# ROPK.gtest.noise, NTE.gtest.noise, ETEN.gtest.noise, ROPKL-v-ROPK.gtest.noise, BPK1.cma.noise, ROP11.cma.noise, ROP16.cma.noise, ROP17.cma.noise, ROP18.cma.noise, ROP19-29-38.cma.noise, ROP20.cma.noise, ROP21-27.cma.noise, ROP22.cma.noise, ROP23.cma.noise, ROP24.cma.noise, ROP25.cma.noise, ROP26.cma.noise, ROP2-8.cma.noise, ROP28.cma.noise, ROP30.cma.noise, ROP31.cma.noise, ROP32.cma.noise, ROP33.cma.noise, ROP34.cma.noise, ROP35.cma.noise, ROP36.cma.noise, ROP37.cma.noise, ROP39.cma.noise, ROP40.cma.noise, ROP41.cma.noise, ROP42-43-44.cma.noise, ROP45.cma.noise, ROP46.cma.noise, ROP4-7.cma.noise, ROP47.cma.noise, ROP48.cma.noise, ROP49.cma.noise, ROP50.cma.noise, ROP5.cma.noise, ROPK-Eten1.cma.noise, ROPK-Eten2a.cma.noise, ROPK-Eten2b.cma.noise, ROPK-Eten3.cma.noise, ROPK-Eten4.cma.noise, ROPK-Eten5.cma.noise, ROPK-Eten6.cma.noise, ROPK-Unique.cma.noise

|  |  |  |  |  |  |  |  |  |  |  |  |  |  |  |  |  |  |  |  |  |  |  |  |  |  |  |  |  |  |  |  |  |  |  |  |  |  |  |  |  |  |  |  |  |  |  |  |  |  |  |
| --- | --- | --- | --- | --- | --- | --- | --- | --- | --- | --- | --- | --- | --- | --- | --- | --- | --- | --- | --- | --- | --- | --- | --- | --- | --- | --- | --- | --- | --- | --- | --- | --- | --- | --- | --- | --- | --- | --- | --- | --- | --- | --- | --- | --- | --- | --- | --- | --- | --- | --- |
|  | \* |  |  |  |  |  |  |  |  |  |  |  |  |  |  |  |  |  |  |  |  |  |  |  |  |  |  |  |  |  |  | \* | \* |  |  |  |  |  |  |  |  |  |  |  |  |  |  |  |  |  |
| ROPK.gtest | L | V | R | G | R | F | L | G | I | G | G | T | G | I | V | F | E | A | T | D | V | E | T | G | E | P | V | A | V | K | V | F | V | T | R | A | K | S | W | S | E | K | E | R | A | P | E | E | E | D |
| NTE.gtest | L | V | R | G | R | P | L | G | S | G | G | W | S | M | V | F | E | A | T | D | Q | E | T | G | E | P | M | A | V | K | V | F | Y | T | W | N | K | - | - | - | - | - | G | E | N | P | S | S | K | D |
| ETEN.gtest | Y | K | I | K | R | Y | L | G | E | G | N | E | S | V | T | L | E | V | V | D | Q | A | T | N | K | P | Y | A | M | R | I | R | T | G | R | P | R | V | H | G | E | P | E | T | A | E | E | L | N | Q |
| ROPKL-v-ROPK.gtest | T | V | M | Q | V | Y | H | P | S | A | E | L | A | A | F | L | H | L | P | E | E | Q | R | E | E | G | V | V | I | K | V | R | P | G | D | P | G | - | - | - | - | - | - | - | - | - | - | - | P | D |
| BPK1.cma | T | T | V | N | V | R | V | P | A | R | E | L | V | R | L | L | S | L | T | P | E | Q | Q | R | E | G | V | S | A | K | V | R | L | I | N | L | L | - | - | - | - | - | - | - | - | - | - | - | - | D |
| ROP11.cma | Y | T | M | E | R | I | L | G | A | G | A | A | S | I | V | I | Q | A | V | Q | E | T | T | R | P | R | V | A | L | R | F | P | V | V | D | V | R | P | E | V | P | E | A | E | L | L | S | Q | L | A |
| ROP16.cma | L | Y | N | R | G | H | L | G | S | G | H | F | G | A | V | I | K | A | S | L | D | D | - | G | T | L | Y | A | A | K | V | P | Y | S | Q | I | V | E | L | E | A | G | I | S | S | A | R | A | E | L |
| ROP17.cma | L | K | K | R | G | F | L | G | G | G | G | F | G | L | V | Y | H | V | E | H | P | T | T | G | Q | P | F | A | L | K | I | F | V | Q | R | V | M | - | - | - | - | - | S | N | K | E | G | D | R | V |
| ROP18.cma | L | V | R | G | A | P | L | G | S | G | G | F | A | T | V | Y | E | A | T | D | V | E | T | N | E | E | L | A | V | K | V | F | M | S | E | K | E | - | - | - | - | - | - | - | - | P | T | D | E | T |
| ROP19-29-38.cma | T | G | P | A | R | F | L | G | M | G | S | T | A | I | V | F | R | L | N | Q | V | K | A | P | T | Q | V | A | A | K | I | C | V | R | R | L | Q | S | W | W | Q | K | K | R | T | L | E | E | I | M |
| ROP20.cma | F | R | R | G | N | P | L | A | S | G | S | Y | N | V | L | S | E | A | T | P | V | P | S | S | E | P | V | A | V | R | I | A | L | S | A | R | S | - | - | - | S | C | E | V | E | A | E | D | E | C |
| ROP21-27.cma | V | I | F | D | D | V | L | G | A | G | G | Q | G | V | V | L | L | A | R | D | V | D | P | G | Q | E | M | A | V | K | I | F | R | I | R | K | D | - | N | R | R | A | S | A | K | H | E | A | R | L |
| ROP22.cma | L | K | G | I | E | L | T | G | A | A | R | G | L | F | V | T | K | V | S | A | V | V | T | K | N | P | L | S | S | S | L | Q | T | R | S | T | Q | F | V | A | E | V | D | K | V | Y | E | E | E | V |
| ROP23.cma | I | T | V | K | D | Y | I | P | V | A | G | W | G | L | C | V | G | V | V | H | P | E | S | N | Q | E | F | L | L | L | N | F | V | S | L | A | D | - | - | - | - | - | - | A | A | W | G | K | W | F |
| ROP24.cma | I | K | Y | D | G | I | L | S | S | G | P | W | S | T | V | L | R | V | M | D | V | D | E | N | Q | E | Y | A | L | K | V | A | R | L | R | A | S | - | - | - | - | - | - | - | L | S | G | A | A | G |
| ROP25.cma | A | I | R | G | K | I | L | G | F | G | A | Y | G | V | V | V | E | F | V | D | V | S | R | S | V | T | Y | A | A | K | I | M | Y | W | S | K | A | K | T | S | A | N | P | E | E | V | Q | Q | T | L |
| ROP26.cma | S | M | R | L | R | P | I | G | I | G | G | T | E | I | L | L | K | M | R | D | K | Q | D | G | G | N | V | P | D | H | I | Y | W | Q | P | L | F | K | E | S | V | D | P | Q | F | L | K | H | G | L |
| ROP2-8.cma | L | V | R | G | T | V | L | G | D | D | D | S | Y | I | C | L | E | A | T | D | Q | E | T | G | E | S | F | E | V | H | V | P | Y | F | T | E | R | - | - | - | - | - | - | - | - | P | P | S | N | A |
| ROP28.cma | I | I | W | H | K | L | L | G | V | G | G | M | G | G | V | L | F | V | E | D | E | D | P | L | K | R | F | A | G | K | I | I | Y | Q | Q | S | E | - | P | S | M | N | L | S | Q | S | K | E | Y | F |
| ROP30.cma | L | V | A | G | R | L | L | G | G | G | S | E | A | M | V | F | A | A | E | E | I | E | L | P | R | L | L | A | I | K | I | Y | L | C | E | A | N | - | L | L | D | Q | P | V | K | A | A | E | E | A |
| ROP31.cma | L | T | R | G | E | L | L | G | C | G | G | T | S | N | V | Y | S | M | T | Q | E | D | - | G | T | K | V | A | V | K | I | L | S | S | Y | A | Q | T | N | L | A | S | V | L | S | A | G | A | S | A |
| ROP32.cma | F | V | R | G | P | L | L | G | V | G | G | N | G | L | V | F | E | A | Q | D | T | D | - | G | H | V | Y | A | L | K | L | L | L | I | R | M | S | S | H | D | A | W | R | R | E | E | D | W | A | F |
| ROP33.cma | M | V | M | L | V | E | T | P | S | K | E | L | R | A | L | V | K | S | A | M | R | A | L | E | E | P | L | V | V | K | A | R | V | F | D | E | R | - | - | - | - | - | - | - | - | - | - | - | - | D |
| ROP34.cma | K | V | T | F | V | D | R | P | S | A | A | L | L | S | F | L | H | L | E | E | E | D | V | P | Y | G | V | V | I | K | A | I | P | Y | D | A | F | - | - | - | - | - | - | - | - | - | - | - | - | D |
| ROP35.cma | L | V | M | I | V | P | H | V | S | E | A | L | A | D | F | L | G | L | D | E | E | T | R | E | H | G | I | V | I | K | G | K | S | S | R | P | G | - | - | - | - | - | - | - | - | - | - | - | C | E |
| ROP36.cma | I | K | R | L | D | M | I | N | Y | G | Y | A | S | A | T | Y | A | V | K | R | I | F | L | D | Q | D | G | A | L | K | V | F | V | V | K | R | N | - | - | - | - | - | - | R | I | E | V | D | L | V |
| ROP37.cma | F | M | R | G | A | F | L | G | A | G | A | T | G | I | V | T | E | L | I | E | K | T | S | G | R | Y | F | A | A | K | F | F | F | K | E | V | A | S | M | E | G | A | S | P | A | A | F | Q | A | A |
| ROP39.cma | L | K | I | R | S | V | A | G | T | G | G | Y | N | I | V | V | K | A | F | V | I | K | E | P | Q | M | V | S | L | R | I | R | M | G | G | A | S | C | Q | V | T | N | E | M | Q | A | A | E | V | V |
| ROP40.cma | F | E | R | G | P | M | L | M | T | D | F | F | A | L | T | V | D | H | Q | - | - | - | - | - | - | P | F | N | M | R | I | F | P | L | P | G | G | - | - | - | F | E | G | E | Q | K | V | E | Q | Y |
| ROP41.cma | M | V | R | G | R | Y | L | G | V | G | G | N | G | I | V | F | E | A | S | T | A | H | S | G | E | S | L | A | L | K | L | L | F | L | D | R | T | F | L | Q | S | P | Q | S | D | I | S | K | A | L |
| ROP42-43-44.cma | V | S | L | R | A | P | L | G | I | G | E | Y | T | A | V | F | S | G | F | I | S | G | I | D | S | E | L | A | V | K | A | F | L | G | K | T | T | - | - | - | - | - | - | - | - | - | - | - | E | S |
| ROP45.cma | L | K | R | G | R | F | L | K | Y | G | G | G | T | L | L | F | E | V | W | E | G | R | - | - | K | R | W | G | L | R | L | H | L | T | Q | L | S | G | V | S | A | W | Q | R | T | V | H | G | V | C |
| ROP46.cma | T | L | I | P | V | F | H | M | P | P | Q | L | T | A | D | L | F | S | P | G | E | S | H | E | S | G | V | M | V | K | V | R | R | G | T | P | G | - | - | - | - | - | - | - | - | - | V | L | P | A |
| ROP4-7.cma | L | V | R | G | R | R | I | G | L | F | R | P | G | M | Q | F | E | A | T | D | Q | A | T | G | E | P | M | T | V | L | V | G | H | T | W | N | K | - | - | - | - | - | - | - | - | P | S | G | K | D |
| ROP47.cma | L | R | M | G | K | V | L | G | G | G | A | R | S | I | V | V | E | V | T | R | V | D | T | G | D | R | W | A | L | K | A | F | L | V | P | R | D | - | - | - | V | I | G | S | P | G | T | P | P | P |
| ROP48.cma | L | Y | R | G | P | V | L | G | R | G | T | R | G | I | A | F | K | G | T | M | V | A | T | G | E | H | F | A | V | K | L | Q | F | V | R | K | S | - | L | F | R | R | R | S | S | L | L | R | D | L |
| ROP49.cma | L | V | R | G | R | N | V | A | V | T | E | G | A | V | L | F | D | V | T | D | N | E | S | K | H | E | Y | T | A | K | L | F | S | V | A | D | K | - | - | - | S | V | V | K | A | L | S | A | V | W |
| ROP50.cma | V | T | R | Q | V | Y | M | G | S | A | K | L | A | E | D | L | R | V | P | H | A | L | L | T | G | G | F | T | V | K | M | C | P | G | S | R | S | - | - | - | - | - | - | - | - | - | - | - | - | K |
| ROP5.cma | L | K | L | V | E | P | L | R | V | G | D | R | S | V | V | F | L | V | R | D | V | E | R | L | E | Y | F | A | L | K | V | F | T | M | G | A | E | - | - | - | - | - | - | - | N | S | R | S | E | L |
| ROPK-Eten1.cma | M | K | V | G | P | A | L | G | V | G | G | A | G | L | V | L | A | V | E | V | L | S | G | A | K | Q | L | A | A | K | I | T | Y | F | P | V | P | P | S | P | Q | E | L | R | H | V | N | H | M | I |
| ROPK-Eten2a.cma | L | K | I | A | K | F | L | G | E | G | T | F | A | L | V | L | E | V | K | D | E | E | T | G | E | V | Y | A | L | R | V | P | F | S | V | N | E | S | E | Y | E | T | E | L | L | E | N | D | V | E |
| ROPK-Eten2b.cma | Y | E | V | T | R | F | L | G | E | G | L | F | S | L | I | L | E | V | K | D | K | E | S | Q | R | F | F | A | L | R | V | P | F | G | A | N | Q | G | E | S | T | E | E | R | L | T | E | A | L | S |
| ROPK-Eten3.cma | V | R | I | C | K | V | V | G | V | G | F | S | T | L | V | V | E | V | E | D | V | E | S | G | G | V | Y | V | M | H | I | P | V | Y | H | K | D | K | F | E | N | S | E | V | F | L | L | E | V | K |
| ROPK-Eten4.cma | L | K | I | T | R | I | I | K | F | G | H | S | S | I | F | V | E | V | Q | D | V | R | T | Y | R | Y | V | T | V | R | I | H | V | C | D | T | K | - | - | - | - | - | - | R | S | Q | P | N | R | T |
| ROPK-Eten5.cma | Y | T | I | K | R | Y | L | Y | E | D | N | E | S | V | T | L | E | I | V | D | Q | A | T | N | L | P | Y | A | M | R | L | R | T | V | R | P | R | V | H | G | E | P | E | T | A | E | E | L | N | Q |
| ROPK-Eten6.cma | Y | V | V | T | R | F | I | D | Q | G | Y | Q | S | V | L | L | E | V | Q | D | R | E | T | E | G | S | A | A | M | R | I | N | F | L | P | N | D | E | N | L | A | L | S | T | A | K | T | S | V | S |
| ROPK-Unique.cma | L | R | R | G | K | V | L | G | S | G | T | F | S | V | V | F | E | G | Q | D | V | K | A | D | Q | E | Y | A | V | K | V | F | V | I | S | Q | S | - | - | - | - | - | - | E | I | S | Q | F | E | T |
|  |  |  |  |  |  |  |  |  |  | 10 |  |  |  |  |  |  |  |  |  | 20 |  |  |  |  |  |  |  |  |  | 30 |  |  |  |  |  |  |  |  |  | 40 |  |  |  |  |  |  |  |  |  | 50 |

|  |  |  |  |  |  |  |  |  |  |  |  |  |  |  |  |  |  |  |  |  |  |  |  |  |  |  |  |  |  |  |  |  |  |  |  |  |  |  |  |  |  |  |  |  |  |  |  |  |  |  |
| --- | --- | --- | --- | --- | --- | --- | --- | --- | --- | --- | --- | --- | --- | --- | --- | --- | --- | --- | --- | --- | --- | --- | --- | --- | --- | --- | --- | --- | --- | --- | --- | --- | --- | --- | --- | --- | --- | --- | --- | --- | --- | --- | --- | --- | --- | --- | --- | --- | --- | --- |
|  |  |  |  |  |  |  |  |  |  |  |  |  |  |  |  |  |  |  |  |  |  |  |  |  | \* | \* | \* | \* |  | \* | \* |  |  |  |  |  | \* |  |  |  |  |  |  |  |  |  |  |  |  |  |
| ROPK.gtest | E | K | F | L | R | E | E | A | S | I | R | R | L | L | P | S | K | N | P | E | E | A | L | R | E | H | G | F | L | V | P | F | D | V | V | R | I | P | G | K | P | D | V | F | R | A | G | E | S | Y |
| NTE.gtest | I | D | Q | L | R | H | E | A | L | A | I | R | L | F | G | V | K | N | P | Y | Q | A | N | R | Y | L | R | F | L | V | P | F | D | L | V | T | I | P | G | K | P | K | M | Q | K | A | R | E | D | G |
| ETEN.gtest | E | Q | L | V | E | E | T | S | A | M | L | Q | A | C | G | S | T | P | L | A | K | A | A | S | E | R | G | L | A | V | P | L | A | V | A | T | I | Q | G | L | P | P | V | L | R | C | - | G | G | V |
| ROPKL-v-ROPK.gtest | P | K | Y | S | V | Y | E | I | Y | A | H | K | W | I | - | - | - | - | - | - | - | L | P | P | K | S | P | F | I | L | P | L | L | G | A | C | R | G | C | - | - | - | - | - | - | - | - | - | - | - |
| BPK1.cma | P | K | Y | S | V | Y | E | P | Y | L | Y | R | E | I | - | - | - | - | - | - | - | L | P | K | R | S | P | L | L | L | P | S | L | G | E | Y | R | G | A | - | - | - | - | - | - | - | - | - | - | - |
| ROP11.cma | E | E | Q | L | R | L | E | I | H | K | A | A | L | V | A | A | G | T | E | G | N | F | D | S | E | W | G | F | A | L | P | F | R | V | G | R | L | S | N | E | G | L | V | L | A | M | D | S | G | K |
| ROP16.cma | V | K | T | I | R | Q | E | L | D | V | R | D | K | L | K | G | L | T | L | T | E | T | V | S | Q | Y | G | L | P | L | C | Q | M | T | L | T | L | P | E | K | A | T | V | V | R | R | G | R | L | F |
| ROP17.cma | S | D | L | I | E | D | E | F | G | V | M | K | Y | F | P | E | W | T | P | A | R | M | Y | S | E | L | R | F | M | V | P | L | L | K | L | R | V | L | G | K | P | E | F | Q | D | V | R | N | H | L |
| ROP18.cma | M | R | D | L | Q | R | E | S | F | C | Y | R | N | F | L | A | K | T | A | K | D | A | Q | E | R | C | R | F | M | V | P | S | D | V | V | M | L | E | G | Q | P | A | S | T | E | V | V | T | T | R |
| ROP19-29-38.cma | E | R | F | L | S | N | E | S | S | V | R | N | A | L | P | H | V | T | P | E | T | M | L | - | E | H | G | L | L | F | P | H | D | V | C | R | V | S | N | M | P | S | W | F | R | A | G | D | S | Y |
| ROP20.cma | L | S | L | A | R | K | Y | S | N | L | M | S | K | L | P | P | F | S | S | R | D | L | L | E | K | F | G | M | V | V | P | Q | L | V | G | Q | L | R | G | R | P | S | L | L | R | K | Q | G | G | N |
| ROP21-27.cma | Q | R | R | V | Q | W | E | T | A | I | W | K | Y | V | R | G | V | S | P | Y | Q | W | S | Q | M | A | H | L | V | M | P | L | D | I | V | E | P | V | E | K | W | E | H | P | D | M | - | E | Q | M |
| ROP22.cma | E | A | R | L | A | W | E | L | R | I | W | R | H | L | P | S | W | R | A | E | Q | L | A | A | E | E | H | I | L | I | P | G | A | L | L | S | H | S | G | R | G | S | P | S | S | R | R | L | S | E |
| ROP23.cma | E | W | R | L | R | K | I | A | E | P | L | Q | W | L | G | L | R | N | P | S | E | A | Y | S | Q | D | R | L | M | L | P | L | D | L | L | E | I | S | N | S | S | R | Y | F | V | R | - | A | P | F |
| ROP24.cma | Y | R | Q | L | L | R | Q | G | L | L | H | H | P | V | - | - | - | S | P | H | H | A | L | W | C | L | Q | L | M | V | A | I | D | L | L | K | L | P | R | I | P | R | F | V | R | E | P | S | R | L |
| ROP25.cma | E | D | F | V | H | E | E | L | E | A | F | K | L | L | T | D | I | S | D | D | D | L | F | Q | K | H | G | L | V | V | P | Q | S | V | R | R | V | A | K | L | P | T | L | L | R | A | S | T | P | L |
| ROP26.cma | V | Q | R | V | Q | K | Q | I | S | L | I | P | P | G | Y | E | G | R | S | E | H | L | C | S | H | Y | G | L | M | F | P | G | G | T | Y | V | L | A | A | D | N | A | P | L | Q | F | D | G | S | R |
| ROP2-8.cma | I | K | Q | M | K | E | E | V | L | R | L | R | L | L | G | I | K | N | Q | K | Q | A | K | V | Y | L | R | F | I | F | P | F | D | L | V | K | D | P | K | K | R | K | M | I | R | V | R | R | D | M |
| ROP28.cma | Y | Q | I | R | N | E | E | I | G | V | A | S | L | F | T | T | T | D | R | M | Q | F | L | F | Q | R | G | F | V | L | P | Q | T | M | F | W | F | E | G | - | - | - | - | T | T | Q | I | E | I | M |
| ROP30.cma | E | A | V | E | W | L | Q | M | A | F | W | R | L | V | P | S | C | V | D | V | L | L | S | R | Q | L | G | V | A | A | P | S | W | V | G | A | V | E | G | M | P | L | V | I | S | G | D | S | H | F |
| ROP31.cma | M | K | I | M | R | R | E | A | G | I | S | E | L | L | G | N | Q | D | L | D | D | I | L | Y | N | D | R | L | L | V | P | S | D | V | L | R | L | P | G | K | N | D | W | F | F | P | E | Q | S | T |
| ROP32.cma | A | R | A | L | R | K | E | M | K | I | L | R | L | F | P | P | D | K | S | P | E | Q | L | Y | E | E | G | F | V | L | P | L | F | Q | G | I | L | A | G | K | P | R | M | T | P | L | T | E | E | F |
| ROP33.cma | G | T | R | V | G | L | E | V | N | G | Q | L | W | A | - | - | - | - | - | - | - | - | A | P | K | N | A | F | I | L | P | A | Y | G | A | W | I | S | D | V | G | D | W | K | P | R | T | Q | K | K |
| ROP34.cma | F | Y | E | S | V | A | E | P | Y | I | H | R | M | F | - | - | - | - | - | - | D | D | P | R | K | F | P | Y | V | V | P | V | L | A | A | L | R | S | T | - | - | - | - | - | - | - | - | - | - | - |
| ROP35.cma | A | K | Q | T | A | W | E | I | N | A | H | Q | N | M | - | - | - | - | - | - | - | - | V | P | Q | N | P | F | I | L | P | L | L | G | A | Y | R | S | R | - | - | - | - | - | - | - | - | - | - | - |
| ROP36.cma | E | E | L | L | L | H | Q | T | R | A | W | R | F | F | P | P | E | R | A | E | Q | L | A | E | T | E | G | L | L | I | P | V | S | L | L | K | L | V | N | D | D | D | E | E | I | G | Q | P | K | G |
| ROP37.cma | Q | Q | R | L | Q | K | E | S | N | G | N | E | L | L | T | T | A | A | Q | V | R | D | L | W | Q | K | G | F | V | A | S | L | G | T | Y | K | L | S | A | - | - | - | - | R | S | G | D | H | Q | L |
| ROP39.cma | Q | K | C | V | S | E | T | D | R | I | M | L | V | F | E | D | V | T | P | Q | V | L | V | S | R | F | R | F | A | V | P | L | F | S | G | R | I | R | H | R | S | A | L | L | G | Q | S | R | G | Y |
| ROP40.cma | K | K | V | M | L | N | E | Q | K | V | L | S | I | F | G | V | A | D | G | K | K | V | S | N | A | Y | H | C | Y | L | P | V | E | E | V | F | F | G | K | G | Q | Q | V | I | S | L | G | P | S | L |
| ROP41.cma | A | V | S | K | R | M | E | M | R | I | R | L | L | L | R | S | V | T | S | A | Q | L | Y | F | R | D | R | F | A | V | P | L | F | C | G | R | I | R | G | L | P | D | L | F | D | I | G | S | R | G |
| ROP42-43-44.cma | M | S | V | A | E | T | E | A | S | I | L | K | I | V | S | L | K | D | P | V | D | A | M | H | R | L | R | L | L | A | P | I | E | T | L | V | Y | R | R | D | G | D | S | A | D | Q | S | E | G | - |
| ROP45.cma | A | E | A | Q | E | L | E | L | H | A | G | R | I | L | Q | N | E | Q | P | P | D | L | F | C | T | E | R | I | A | L | P | I | A | V | G | E | V | S | S | L | P | K | V | T | Q | I | R | D | D | E |
| ROP46.cma | S | D | A | G | I | Y | E | A | F | A | H | K | E | L | - | - | - | - | - | - | - | - | A | P | K | S | P | F | V | L | P | L | L | G | A | C | Q | G | C | - | - | - | - | - | - | - | - | - | - | - |
| ROP4-7.cma | L | D | K | L | R | H | Q | A | L | A | I | G | L | F | K | V | K | N | P | Y | L | A | N | R | Y | L | R | F | L | A | P | F | D | L | V | T | I | P | G | K | P | L | V | Q | K | A | K | E | V | G |
| ROP47.cma | W | D | A | I | S | E | E | A | H | A | I | K | Q | L | P | H | T | V | P | A | V | V | Y | R | N | L | R | F | L | V | P | T | D | V | L | I | Y | E | G | D | G | M | E | K | L | W | N | T | G | S |
| ROP48.cma | V | K | Y | L | Q | Q | E | F | A | V | R | N | M | L | V | A | A | Y | G | E | E | N | V | Q | K | K | G | M | V | L | P | L | T | V | R | R | R | H | A | F | G | D | F | I | T | T | P | S | F | T |
| ROP49.cma | K | S | F | K | E | K | N | V | E | S | V | L | V | E | - | R | F | G | S | D | E | N | V | L | K | S | G | F | L | V | P | V | G | G | G | S | I | V | N | V | P | P | V | L | Q | P | A | S | D | T |
| ROP50.cma | P | W | K | A | L | T | E | I | F | L | R | K | K | L | - | - | - | - | - | - | - | L | P | P | N | S | P | F | F | V | P | L | V | A | T | A | K | G | K | - | - | - | - | - | - | - | - | - | - | - |
| ROP5.cma | E | R | L | H | E | A | T | F | A | A | A | R | L | L | E | S | - | - | P | E | E | A | R | D | R | R | R | L | L | L | P | S | D | A | V | A | V | Q | S | Q | P | P | F | A | Q | L | S | S | D | Y |
| ROPK-Eten1.cma | E | E | L | F | R | Q | E | L | D | P | L | H | T | V | G | A | A | S | S | K | K | V | A | R | Q | N | Q | W | V | V | P | L | F | V | A | T | V | G | S | P | K | A | S | L | Q | V | H | Q | N | T |
| ROPK-Eten2a.cma | H | Q | V | L | A | E | E | K | G | L | R | A | I | L | G | K | M | P | A | D | K | A | A | A | R | M | G | L | A | V | P | L | A | T | A | Q | L | D | K | L | P | N | G | L | H | S | - | N | G | V |
| ROPK-Eten2b.cma | E | L | L | L | A | E | E | E | G | I | R | T | A | L | D | K | T | P | A | A | K | G | A | S | H | R | G | I | A | V | P | L | A | T | A | E | L | E | D | M | P | A | D | L | Y | A | - | E | G | L |
| ROPK-Eten3.cma | E | A | M | V | A | E | E | D | A | V | R | Q | A | C | G | N | V | P | A | E | L | A | A | S | Q | K | G | I | A | V | P | L | Y | T | A | E | I | Q | S | L | D | E | L | H | S | R | - | E | D | F |
| ROPK-Eten4.cma | K | A | V | V | E | E | T | Q | S | T | T | L | A | R | G | G | T | D | M | D | L | A | T | S | H | S | G | L | L | V | P | L | F | T | A | S | L | E | G | L | A | A | E | T | V | C | - | G | N | Y |
| ROPK-Eten5.cma | R | S | L | V | E | T | T | S | S | M | L | Q | A | I | G | E | S | D | L | R | D | A | A | E | E | R | G | L | A | V | I | S | A | V | A | T | I | Q | G | V | P | T | V | M | R | C | - | T | H | V |
| ROPK-Eten6.cma | P | Q | M | I | R | V | A | Q | A | S | L | G | I | S | G | S | T | P | L | A | S | A | A | Q | E | K | G | I | A | P | T | R | F | M | A | R | I | D | G | F | P | K | I | L | R | Y | - | G | G | L |
| ROPK-Unique.cma | E | E | R | L | K | R | E | F | K | I | R | R | L | A | Q | D | Y | D | E | D | S | L | A | A | K | Q | G | L | L | V | P | L | C | V | G | R | V | T | G | A | P | A | E | L | T | V | G | K | S | L |
|  |  |  |  |  |  |  |  |  |  | 60 |  |  |  |  |  |  |  |  |  | 70 |  |  |  |  |  |  |  |  |  | 80 |  |  |  |  |  |  |  |  |  | 90 |  |  |  |  |  |  |  |  |  | 100 |

|  |  |  |  |  |  |  |  |  |  |  |  |  |  |  |  |  |  |  |  |  |  |  |  |  |  |  |  |  |  |  |  |  |  |  |  |  |  |  |  |  |  |  |  |  |  |  |  |  |  |  |
| --- | --- | --- | --- | --- | --- | --- | --- | --- | --- | --- | --- | --- | --- | --- | --- | --- | --- | --- | --- | --- | --- | --- | --- | --- | --- | --- | --- | --- | --- | --- | --- | --- | --- | --- | --- | --- | --- | --- | --- | --- | --- | --- | --- | --- | --- | --- | --- | --- | --- | --- |
|  |  |  |  |  |  |  |  | \* |  | \* | \* |  |  |  |  |  |  |  |  |  |  |  |  | \* |  |  |  |  |  | \* |  |  |  |  |  |  |  |  |  |  | \* | \* |  | \* |  |  |  |  |  |  |
| ROPK.gtest | W | V | L | N | V | V | F | L | Y | P | R | M | Q | C | D | L | E | D | L | V | E | S | L | L | P | L | S | D | A | A | R | L | Y | L | T | R | Q | M | I | R | L | V | A | H | L | H | D | Y | G | V |
| NTE.gtest | W | V | I | N | Y | F | F | L | Y | P | R | A | Q | V | D | M | E | T | F | V | E | E | L | Y | P | L | A | D | A | A | R | L | Y | L | T | V | Q | A | I | R | L | V | A | H | L | Q | H | E | G | V |
| ETEN.gtest | Y | V | M | N | D | V | E | L | T | E | R | V | S | G | R | L | Q | D | I | F | D | A | L | Q | R | M | P | T | E | A | K | E | Y | I | A | R | R | V | L | L | Q | V | L | H | L | Q | Q | A | G | W |
| ROPKL-v-ROPK.gtest | T | T | F | N | T | Y | M | F | H | P | R | T | R | G | D | V | R | D | Y | L | C | Q | R | P | - | S | S | C | V | D | V | Q | L | A | A | A | E | M | V | A | A | V | K | Q | L | H | R | L | G | F |
| BPK1.cma | - | - | F | L | T | Y | I | F | H | P | L | S | K | G | L | V | G | D | M | L | E | T | G | R | - | - | S | H | P | D | V | Q | V | L | A | A | N | M | V | A | A | L | K | S | L | H | N | L | G | L |
| ROP11.cma | A | I | L | N | F | V | T | V | S | P | A | M | M | C | D | L | H | T | L | R | S | S | S | R | - | - | T | P | A | M | D | E | F | V | V | M | R | V | L | Q | L | A | A | N | L | E | R | L | Q | L |
| ROP16.cma | V | V | S | K | E | V | M | L | L | P | L | I | D | S | A | L | N | S | L | V | Q | S | Q | P | - | - | P | F | L | F | Q | R | A | V | A | R | E | A | I | L | A | L | A | K | L | H | E | L | G | F |
| ROP17.cma | R | I | Y | S | V | C | A | L | F | P | K | A | Q | G | D | L | E | E | A | V | V | L | L | A | T | N | A | Y | N | I | R | M | S | C | T | I | Q | M | V | K | L | L | A | R | F | H | A | F | E | L |
| ROP18.cma | W | V | P | N | Y | F | L | L | M | M | R | A | E | T | D | M | S | K | V | I | S | W | V | F | E | F | G | L | V | V | R | M | Y | L | S | S | Q | A | I | K | L | V | A | N | V | Q | A | Q | G | I |
| ROP19-29-38.cma | F | V | L | P | V | V | A | L | F | P | V | F | T | C | D | L | Q | Q | V | V | Q | S | G | L | - | - | S | R | A | A | K | L | Y | I | T | R | Q | L | L | K | S | V | A | W | L | H | R | N | G | V |
| ROP20.cma | S | L | L | N | F | V | Q | I | M | P | L | M | A | C | D | L | N | Q | F | R | I | T | D | P | - | - | - | - | N | A | V | K | F | V | V | K | R | M | I | Q | L | L | A | L | F | G | A | A | G | L |
| ROP21-27.cma | R | Y | W | H | Q | W | L | I | F | D | S | F | A | G | D | V | A | R | M | K | K | I | M | S | - | G | S | G | S | V | R | L | E | V | T | K | Q | M | F | M | A | C | M | R | L | H | D | M | G | M |
| ROP22.cma | K | V | F | N | V | F | I | W | Y | P | A | G | Y | R | D | T | L | E | S | S | M | M | H | S | S | T | P | R | D | V | M | E | S | I | T | V | Q | M | I | L | A | V | A | N | L | N | S | Y | G | L |
| ROP23.cma | V | V | P | N | L | F | V | L | M | P | R | P | A | T | S | L | S | D | L | V | S | F | M | S | G | Q | T | L | A | V | R | L | S | L | T | L | Q | T | I | R | V | V | A | G | F | N | N | R | G | F |
| ROP24.cma | T | I | A | N | R | F | L | L | M | P | L | A | Y | T | D | L | L | A | V | L | R | A | L | F | P | G | G | F | L | A | R | L | H | M | T | A | E | V | V | T | I | V | A | N | Y | H | K | Q | G | F |
| ROP25.cma | F | F | H | N | Q | L | I | M | Y | P | A | L | K | C | S | L | D | M | M | A | Q | D | H | F | - | - | T | R | E | G | L | R | V | L | V | R | S | L | V | K | V | V | A | G | L | H | Q | L | G | F |
| ROP26.cma | A | V | L | N | L | F | I | Q | F | S | P | A | E | I | P | L | S | V | L | S | Y | K | N | L | - | - | S | P | T | L | A | A | F | L | V | K | Q | M | I | L | N | T | A | M | L | H | S | M | G | V |
| ROP2-8.cma | W | V | L | S | R | F | F | L | Y | P | R | M | Q | S | N | L | Q | I | L | G | D | V | L | L | S | L | V | H | H | A | R | L | Q | L | T | L | Q | L | I | R | L | A | A | S | L | H | H | Y | G | L |
| ROP28.cma | R | L | D | P | H | V | I | A | Y | P | I | V | G | P | D | L | E | N | L | E | P | P | E | W | - | - | N | L | S | T | V | T | Y | I | I | Y | K | L | T | H | L | M | A | T | M | Q | E | M | N | L |
| ROP30.cma | A | I | L | N | R | I | S | A | M | P | M | M | L | G | D | L | L | A | V | S | P | T | A | L | - | - | R | V | A | D | R | I | Y | L | I | L | R | L | I | Q | V | V | A | N | L | H | C | L | G | V |
| ROP31.cma | V | I | Y | N | Y | V | V | - | - | P | L | L | D | T | T | - | - | - | - | - | - | - | - | - | Q | K | S | F | A | A | R | V | S | A | T | V | Q | M | V | K | V | V | A | G | L | H | R | K | G | L |
| ROP32.cma | G | A | T | S | V | V | V | G | F | H | K | V | A | C | S | V | G | Q | L | F | S | A | H | R | - | L | P | D | G | V | K | L | E | L | T | K | Q | M | V | D | R | V | A | R | L | H | S | Y | G | I |
| ROP33.cma | K | L | F | M | A | F | M | V | M | P | R | T | R | G | D | V | R | D | Y | L | C | K | R | S | - | - | N | P | V | D | V | K | Y | A | A | A | E | M | L | Y | S | V | Q | Q | L | H | R | E | G | F |
| ROP34.cma | S | K | R | V | L | Y | L | V | L | P | L | Y | R | - | E | L | P | E | T | V | D | E | E | A | - | - | R | S | L | D | F | V | L | L | L | A | E | M | A | M | A | V | C | Q | L | H | E | R | N | L |
| ROP35.cma | S | S | E | N | V | Y | L | V | L | P | R | A | R | G | D | V | S | Q | Y | V | R | A | A | P | - | - | D | S | V | N | V | R | L | A | A | A | E | M | A | Y | A | V | Y | L | L | H | R | H | G | F |
| ROP36.cma | D | I | L | N | A | F | L | L | Y | P | R | A | Y | C | S | L | K | F | F | L | I | S | W | G | T | A | P | M | K | A | M | I | S | I | S | I | Q | M | V | Y | A | V | A | S | L | N | S | Y | G | L |
| ROP37.cma | M | V | L | S | D | F | L | L | L | P | L | M | G | P | R | L | D | S | L | P | H | T | A | L | - | - | S | D | N | A | Q | K | Y | L | F | H | T | L | V | T | V | V | G | S | L | H | K | S | G | L |
| ROP39.cma | A | L | L | N | Y | M | D | V | L | P | P | M | L | C | D | L | F | A | A | Q | S | V | V | Q | - | - | - | P | S | M | T | R | A | I | A | K | Q | L | L | E | L | V | A | H | L | Q | A | L | G | V |
| ROP40.cma | K | M | P | S | V | V | F | L | Y | P | S | A | S | A | T | L | G | Q | V | A | E | A | I | K | L | V | A | Q | A | A | Q | L | H | I | T | L | Q | L | I | K | L | V | A | I | T | T | S | K | G | I |
| ROP41.cma | A | G | C | S | I | V | V | L | Y | K | R | L | Y | C | S | L | A | D | L | L | S | A | C | A | S | L | S | Y | Q | A | R | L | F | L | T | K | Q | M | I | E | V | V | H | N | L | H | C | K | G | L |
| ROP42-43-44.cma | - | - | - | R | P | V | V | L | V | P | K | A | S | S | S | L | V | D | V | I | N | F | L | R | R | A | K | K | I | V | R | L | G | A | T | V | Q | L | I | Q | L | L | A | A | L | H | T | R | K | V |
| ROP45.cma | H | V | T | N | I | G | G | L | Y | P | L | A | A | C | D | M | R | E | V | V | E | Q | Y | H | - | M | P | E | E | V | K | V | E | A | V | R | Q | M | V | G | A | V | G | R | L | H | S | R | G | V |
| ROP46.cma | T | T | G | N | V | Y | M | F | T | P | L | L | Q | G | D | I | R | R | V | A | V | Q | H | P | - | S | Q | C | I | S | G | E | L | V | L | K | E | M | A | A | S | L | K | V | L | H | D | A | G | L |
| ROP4-7.cma | W | V | I | N | L | L | F | L | L | P | P | T | H | V | D | M | E | R | F | V | E | E | L | Y | P | L | A | D | A | A | R | L | Y | L | T | V | Q | A | V | R | L | V | A | H | L | Q | D | E | G | V |
| ROP47.cma | N | F | Y | T | T | Y | L | L | F | P | I | A | Q | G | S | L | L | D | A | V | R | A | M | F | A | R | T | L | A | A | N | V | S | F | S | I | Q | I | I | R | L | T | A | L | L | H | S | H | G | V |
| ROP48.cma | M | F | W | N | F | L | V | F | Y | P | L | M | K | C | T | L | G | N | L | L | V | G | P | H | - | W | P | R | E | A | R | L | F | V | A | K | R | L | I | E | I | A | A | A | L | Q | R | A | G | V |
| ROP49.cma | S | F | L | G | A | L | I | L | Y | P | R | V | A | Q | T | L | D | A | L | L | W | S | G | A | T | L | E | K | A | A | K | F | F | L | I | R | R | L | I | R | S | V | G | D | L | H | R | S | E | L |
| ROP50.cma | - | E | A | A | T | L | Q | F | F | P | P | T | R | G | S | L | R | A | I | A | T | Q | V | P | - | E | K | L | N | V | - | V | L | V | M | A | E | M | V | A | A | V | E | A | L | H | G | L | G | I |
| ROP5.cma | A | V | A | N | Y | F | L | L | M | P | A | A | S | V | D | L | E | L | L | F | R | T | L | D | D | E | G | I | L | A | R | H | I | L | T | A | Q | L | I | R | L | A | A | N | L | Q | S | K | G | L |
| ROPK-Eten1.cma | V | F | G | N | K | V | L | L | S | E | V | M | L | G | D | G | A | D | L | M | H | E | S | P | R | L | P | V | E | A | R | E | Q | V | C | L | Q | V | I | E | T | V | A | R | L | H | A | T | G | W |
| ROPK-Eten2a.cma | F | I | S | S | K | V | Q | L | T | E | R | F | V | V | H | L | E | D | F | I | D | Q | V | P | T | L | P | T | D | A | K | T | Y | I | A | Q | R | L | L | L | Q | V | L | H | L | Q | E | R | G | F |
| ROPK-Eten2b.cma | V | V | S | N | K | V | Q | L | T | E | L | F | Y | G | D | L | E | T | L | L | E | H | S | P | L | L | P | L | D | A | K | I | Y | I | A | Q | R | L | L | L | Q | V | L | H | L | Q | E | I | G | A |
| ROPK-Eten3.cma | Y | I | F | N | R | V | E | L | M | E | K | V | D | G | S | L | A | Q | L | R | S | E | A | P | S | V | M | T | E | A | R | D | Y | I | A | S | R | L | L | H | I | V | L | K | L | E | Q | S | G | V |
| ROPK-Eten4.cma | V | V | L | K | N | A | E | A | Y | E | E | L | V | G | D | L | D | K | T | L | P | T | L | R | W | D | A | N | L | G | I | N | Y | V | A | R | T | L | V | I | E | T | L | N | L | Q | R | L | G | V |
| ROPK-Eten5.cma | Y | L | V | N | D | V | E | I | G | E | R | Y | S | G | R | L | S | D | I | F | I | P | E | N | R | M | P | D | N | A | K | E | Y | M | A | K | R | M | L | L | Q | V | L | Q | L | Q | R | A | G | F |
| ROPK-Eten6.cma | S | V | M | S | A | V | E | L | T | E | N | V | E | V | S | L | Q | D | A | L | D | A | L | Q | L | P | P | T | G | T | K | A | Y | I | A | R | S | V | L | K | T | V | L | H | M | Q | Q | A | G | W |
| ROPK-Unique.cma | H | I | F | N | H | V | S | L | Y | P | R | L | H | G | S | L | Q | D | L | W | S | K | W | L | - | - | T | P | X | A | K | L | Y | L | T | F | Q | L | V | T | T | V | A | Y | L | H | R | I | N | L |
|  |  |  |  |  |  |  |  |  |  | 110 |  |  |  |  |  |  |  |  |  | 120 |  |  |  |  |  |  |  |  |  | 130 |  |  |  |  |  |  |  |  |  | 140 |  |  |  |  |  |  |  |  |  | 150 |

|  |  |  |  |  |  |  |  |  |  |  |  |  |  |  |  |  |  |  |  |  |  |  |  |  |  |  |  |  |  |  |  |  |  |  |  |  |  |  |  |  |  |  |  |  |  |  |  |  |  |  |
| --- | --- | --- | --- | --- | --- | --- | --- | --- | --- | --- | --- | --- | --- | --- | --- | --- | --- | --- | --- | --- | --- | --- | --- | --- | --- | --- | --- | --- | --- | --- | --- | --- | --- | --- | --- | --- | --- | --- | --- | --- | --- | --- | --- | --- | --- | --- | --- | --- | --- | --- |
|  |  |  | \* |  |  |  |  |  |  | \* |  |  |  |  |  |  |  |  | \* |  |  |  |  | \* | \* | \* |  | \* |  | \* |  |  |  |  |  |  |  |  |  |  |  |  |  | \* |  |  |  |  |  |  |
| ROPK.gtest | V | H | G | D | I | K | P | E | N | F | L | L | S | R | D | G | S | V | Y | L | G | D | F | G | S | A | V | R | I | G | E | R | R | S | V | A | S | G | T | P | G | Y | A | P | P | E | T | A | A | C |
| NTE.gtest | V | H | G | D | I | Q | P | D | N | F | C | L | K | R | E | G | G | L | F | L | G | D | F | G | S | L | V | R | A | G | T | R | K | S | P | A | V | V | T | R | G | Y | A | P | P | E | T | R | T | A |
| ETEN.gtest | S | H | N | G | L | K | C | R | N | F | F | V | R | E | D | G | S | F | L | L | G | G | F | G | S | G | T | P | F | G | E | V | I | R | N | V | G | I | D | P | R | Y | T | E | P | E | L | G | A | N |
| ROPKL-v-ROPK.gtest | L | H | R | D | I | K | L | D | N | F | F | V | G | R | D | G | H | V | V | L | A | D | F | D | T | A | W | P | I | G | T | P | A | S | W | L | V | G | T | S | G | Y | T | A | P | E | I | D | R | N |
| BPK1.cma | L | H | R | S | I | E | L | N | S | F | S | V | L | P | D | G | T | V | V | L | G | G | L | Q | T | A | A | P | I | G | T | E | T | V | W | A | G | F | F | G | Q | E | T | A | P | E | I | D | R | N |
| ROP11.cma | T | H | L | D | I | T | D | E | N | V | L | V | G | R | D | G | Q | L | F | L | G | G | F | Q | H | V | R | A | K | G | G | I | S | C | G | K | I | P | S | V | T | F | T | D | P | R | L | A | H | C |
| ROP16.cma | A | H | G | D | V | K | L | N | N | M | M | I | D | V | H | G | F | G | H | M | L | D | M | G | S | V | R | P | V | D | S | - | - | C | V | S | E | E | D | K | Y | Y | W | A | P | E | L | A | K | S |
| ROP17.cma | V | H | G | D | V | K | L | Q | N | F | L | V | D | K | S | G | L | L | L | L | S | D | F | T | Q | I | L | R | T | N | E | R | R | Y | P | P | V | V | T | V | I | Y | M | S | P | E | I | A | T | C |
| ROP18.cma | V | H | T | D | I | K | P | A | N | F | L | L | L | K | D | G | R | L | F | L | G | D | F | G | T | Y | R | I | N | N | S | - | V | G | R | A | I | G | T | P | G | Y | E | P | P | E | R | P | - | - |
| ROP19-29-38.cma | A | H | N | D | L | K | L | E | N | V | L | L | S | A | E | G | K | A | V | I | G | D | F | G | F | A | V | K | L | G | T | - | S | S | A | I | Q | F | T | A | P | Y | L | D | P | Q | T | A | E | A |
| ROP20.cma | V | H | Q | D | I | K | T | E | N | F | L | V | S | R | Q | G | R | L | Y | L | A | D | F | D | A | V | V | R | E | N | D | T | I | C | N | K | K | L | S | L | L | F | S | P | P | E | V | L | R | C |
| ROP21-27.cma | V | H | S | D | I | K | L | P | N | Y | F | I | S | S | D | G | R | I | F | L | G | D | H | S | L | A | R | P | I | G | E | N | S | P | C | M | W | G | T | L | R | Y | L | P | P | E | N | M | K | C |
| ROP22.cma | V | H | T | H | L | R | S | S | S | F | V | F | T | P | E | G | I | V | L | L | S | D | L | N | R | V | V | R | Q | G | E | F | M | R | E | A | S | L | N | D | L | T | A | S | P | E | E | R | V | C |
| ROP23.cma | I | D | A | D | I | R | P | A | S | F | F | V | N | A | K | G | L | V | F | L | G | I | F | T | R | T | T | I | A | K | R | G | - | R | R | A | S | G | R | R | Q | F | T | A | P | E | L | V | F | - |
| ROP24.cma | V | H | G | D | I | Q | P | Q | K | M | L | I | M | P | N | G | S | V | V | L | G | G | F | L | D | F | R | T | P | G | A | R | R | S | P | D | V | F | T | V | P | Y | L | P | P | E | H | L | - | - |
| ROP25.cma | T | H | N | D | I | K | P | Q | N | F | L | V | G | G | D | G | M | V | Y | V | G | D | F | A | Y | L | I | P | I | D | E | - | I | C | N | K | G | F | T | I | D | Y | S | S | P | E | L | M | A | C |
| ROP26.cma | V | H | N | G | I | S | S | A | A | F | F | L | S | G | N | R | L | L | Y | L | G | N | F | G | A | A | R | F | Y | T | G | K | P | F | E | E | A | G | H | V | M | Y | Y | D | P | E | T | A | S | V |
| ROP2-8.cma | V | H | A | D | F | Q | V | R | N | I | L | L | D | Q | R | G | G | V | F | L | T | G | F | E | H | L | V | R | D | G | A | - | S | S | P | - | - | I | G | R | G | F | A | P | P | E | T | T | - | A |
| ROP28.cma | V | H | T | D | V | K | A | E | N | F | L | A | R | D | D | G | E | L | F | V | A | D | L | S | M | S | V | K | M | A | T | L | I | P | C | F | Q | G | T | M | S | Y | L | D | P | N | V | A | E | C |
| ROP30.cma | L | H | N | D | L | K | L | E | N | L | L | I | G | L | D | G | Q | L | Y | V | G | D | L | G | A | I | L | P | N | T | G | - | R | F | Q | R | I | G | T | D | V | Y | L | D | P | Q | S | A | A | D |
| ROP31.cma | V | H | G | D | L | K | P | S | N | F | L | V | S | N | S | G | I | V | L | L | G | D | F | S | Y | A | Y | V | R | G | Q | - | - | - | - | - | - | - | - | - | - | - | M | S | T | E | N | I | R | M |
| ROP32.cma | L | H | G | D | V | K | W | E | N | F | F | L | D | D | N | G | R | V | F | L | G | D | F | E | Q | A | Q | S | L | G | H | R | Q | T | G | P | - | - | - | - | - | - | C | G | P | R | R | A | A | C |
| ROP33.cma | L | H | R | D | I | K | L | T | N | F | F | V | G | Y | D | G | H | V | L | L | A | D | F | D | G | V | W | P | I | G | V | P | A | K | Y | L | V | Y | T | R | G | Y | L | A | P | E | I | D | P | H |
| ROP34.cma | A | H | R | D | L | K | E | D | N | F | L | V | S | P | E | G | H | I | V | V | S | D | L | A | T | L | D | I | T | D | N | K | - | S | F | L | I | G | T | S | G | Y | M | P | P | E | T | R | S | S |
| ROP35.cma | L | H | R | D | I | K | P | H | N | F | F | V | G | F | D | G | H | V | V | L | A | D | F | E | G | V | W | P | K | G | M | W | V | T | E | V | V | Y | T | R | G | Y | L | A | P | E | L | R | R | D |
| ROP36.cma | V | H | G | H | I | R | P | S | S | F | V | M | T | T | D | G | K | V | L | L | T | D | F | H | T | V | V | E | E | G | S | K | L | R | V | R | A | M | A | D | L | N | A | S | P | E | E | L | E | C |
| ROP37.cma | V | H | G | N | I | R | T | T | N | V | L | I | N | K | T | G | E | A | G | L | A | D | F | S | L | L | M | Q | T | G | S | D | V | D | T | M | S | F | H | P | T | Q | S | E | P | E | R | V | H | S |
| ROP39.cma | V | H | S | D | L | K | P | E | N | V | L | V | D | E | A | G | N | L | F | L | A | D | F | D | K | A | H | K | V | G | S | R | V | C | G | S | L | P | H | S | L | F | A | D | P | Q | T | V | I | C |
| ROP40.cma | L | L | R | R | L | S | L | E | N | F | L | L | R | G | D | G | V | V | F | L | S | G | F | S | E | L | V | N | E | K | E | I | F | E | T | E | D | G | T | L | V | T | Q | P | P | T | R | - | - | - |
| ROP41.cma | V | H | R | D | I | K | D | S | N | F | F | L | D | E | Q | G | N | I | Y | L | G | D | F | G | L | S | V | P | N | G | S | - | T | E | A | L | V | Y | T | P | K | L | T | D | P | S | D | V | G | I |
| ROP42-43-44.cma | V | H | G | K | L | E | P | K | S | V | L | L | F | S | H | G | L | L | Y | L | S | D | L | G | R | A | R | R | H | G | E | - | R | F | T | T | S | R | P | S | R | Y | G | A | P | E | V | - | - | - |
| ROP45.cma | V | H | L | D | L | K | L | E | H | F | L | L | D | N | H | G | H | V | Y | L | G | D | L | S | T | S | E | K | I | I | G | V | N | S | C | E | F | G | S | L | P | F | L | S | P | E | H | L | A | C |
| ROP46.cma | V | H | G | D | V | K | E | Q | N | F | F | V | G | R | D | G | H | V | V | I | A | D | F | G | A | L | G | Y | P | G | C | - | - | S | L | E | A | G | T | L | G | Y | I | A | P | E | R | L | - | - |
| ROP4-7.cma | V | H | G | K | I | L | P | D | S | F | C | L | K | R | E | G | G | L | Y | L | R | D | F | G | S | L | V | R | A | G | T | - | K | V | V | A | P | N | A | Q | Q | F | S | P | P | E | V | R | A | R |
| ROP47.cma | V | H | G | D | F | Q | T | K | S | F | F | L | C | Q | D | G | S | L | F | L | G | R | V | R | S | L | T | R | V | G | A | P | Y | S | S | A | T | D | S | P | R | C | T | P | P | E | V | L | - | - |
| ROP48.cma | L | H | R | D | I | F | E | S | K | I | L | L | Q | E | S | G | D | I | F | F | A | D | F | G | A | S | G | K | H | - | - | - | - | - | - | M | Q | S | G | I | A | W | I | E | L | Q | L | - | - | - |
| ROP49.cma | C | H | G | N | L | H | P | T | N | I | V | I | L | A | S | G | D | I | A | L | T | S | F | G | S | C | F | Q | S | - | - | - | - | - | - | - | - | - | - | - | L | F | V | L | S | D | T | - | - | - |
| ROP50.cma | L | H | G | A | L | S | L | D | A | F | Y | V | N | R | E | G | H | V | Q | L | S | D | F | D | S | A | I | V | D | - | - | - | - | N | V | F | R | P | I | S | E | M | A | E | P | V | R | D | G | Y |
| ROP5.cma | V | H | G | R | F | T | P | D | N | L | F | I | M | P | D | G | R | L | M | L | G | D | V | S | A | L | R | K | V | G | T | - | R | S | S | - | - | V | P | V | T | Y | A | P | R | E | F | L | S | N |
| ROPK-Eten1.cma | C | H | A | D | I | K | P | E | N | F | L | I | S | K | S | G | K | V | H | L | A | D | F | G | M | A | G | E | A | G | D | - | R | C | T | E | K | I | T | P | M | F | M | D | P | S | H | A | E | C |
| ROPK-Eten2a.cma | C | H | N | D | L | K | L | D | G | C | L | M | R | A | D | G | S | F | L | L | G | D | L | G | S | S | S | P | A | G | V | E | I | S | L | V | G | T | T | P | E | Y | A | E | P | E | F | F | M | N |
| ROPK-Eten2b.cma | C | H | N | D | L | K | L | S | S | C | F | M | R | A | D | G | S | F | L | L | G | D | F | A | S | I | N | V | A | G | T | E | A | K | F | V | G | A | T | L | A | F | T | E | P | E | L | F | M | N |
| ROPK-Eten3.cma | G | H | N | K | I | D | W | S | S | F | F | L | R | P | D | G | S | F | L | L | G | N | F | S | S | A | S | P | F | G | K | P | T | N | L | F | S | S | M | S | D | Y | P | E | P | Q | M | M | L | Q |
| ROPK-Eten4.cma | S | H | N | R | L | E | W | A | N | L | F | V | N | T | D | G | T | V | L | L | G | G | L | D | A | V | T | R | F | G | D | S | L | L | S | A | R | M | N | P | R | F | V | E | P | Q | L | M | A | D |
| ROPK-Eten5.cma | S | H | N | N | L | H | L | G | N | F | F | M | R | I | D | G | S | C | L | L | T | N | F | G | A | G | T | M | I | G | E | R | L | R | V | E | A | V | D | P | R | Y | A | E | M | E | L | G | A | A |
| ROPK-Eten6.cma | S | H | N | G | L | N | C | R | S | F | G | V | Q | E | D | G | S | V | L | L | L | G | L | Q | S | S | V | P | F | G | E | V | I | D | N | V | G | I | S | P | L | T | T | E | P | E | L | L | A | N |
| ROPK-Unique.cma | V | H | R | D | L | K | P | A | N | F | F | M | D | S | D | G | R | V | F | L | G | D | F | G | F | V | A | P | I | G | E | N | V | C | V | G | N | G | T | P | H | Y | V | D | P | E | L | A | K | C |
|  |  |  |  |  |  |  |  |  |  | 160 |  |  |  |  |  |  |  |  |  | 170 |  |  |  |  |  |  |  |  |  | 180 |  |  |  |  |  |  |  |  |  | 190 |  |  |  |  |  |  |  |  |  | 200 |

|  |  |  |  |  |  |  |  |  |  |  |  |  |  |  |  |  |  |  |  |  |  |  |  |  |  |  |  |  |  |  |  |  |  |  |  |  |  |  |  |  |  |  |  |  |  |  |  |  |  |  |
| --- | --- | --- | --- | --- | --- | --- | --- | --- | --- | --- | --- | --- | --- | --- | --- | --- | --- | --- | --- | --- | --- | --- | --- | --- | --- | --- | --- | --- | --- | --- | --- | --- | --- | --- | --- | --- | --- | --- | --- | --- | --- | --- | --- | --- | --- | --- | --- | --- | --- | --- |
|  |  |  |  |  |  |  |  |  |  |  |  |  |  |  |  |  | \* |  | \* |  |  |  |  |  |  |  |  | \* |  | \* | \* |  |  |  |  |  |  |  |  |  |  |  |  |  |  |  |  |  |  |  |
| ROPK.gtest | A | R | R | Y | G | F | G | K | T | E | T | T | Y | T | E | K | R | D | A | W | A | L | G | V | T | L | Y | Q | I | W | C | G | R | L | P | F | G | L | S | E | Y | A | Y | D | D | E | P | P | A | Q |
| NTE.gtest | A | R | T | M | - | F | Q | D | R | T | T | T | M | T | F | A | F | D | A | W | Q | L | G | L | T | I | Y | W | I | W | C | G | D | L | P | F | G | L | M | G | E | E | Y | S | P | E | F | P | T | G |
| ETEN.gtest | A | W | C | Y | E | D | C | K | G | H | A | V | V | N | A | K | S | D | M | W | S | L | G | I | C | L | Y | Q | I | F | T | D | E | L | P | F | G | L | S | D | I | P | S | S | D | E | S | A | I | P |
| ROPKL-v-ROPK.gtest | F | L | L | D | R | A | L | N | T | Q | S | R | H | T | E | K | S | D | V | Y | S | L | G | V | A | F | R | N | L | A | Q | L | N | G | P | M | - | - | - | - | - | - | - | - | - | - | - | - | - | - |
| BPK1.cma | F | L | A | D | F | A | L | - | T | Q | S | R | H | T | V | K | S | D | V | Y | S | L | G | V | A | F | R | N | L | V | Q | L | N | G | N | M | G | P | D | D | R | G | I | V | - | - | - | - | - | - |
| ROP11.cma | A | V | N | - | - | N | P | G | S | Y | A | A | V | N | P | A | V | D | A | W | M | A | G | M | M | L | L | R | W | F | C | G | D | V | F | F | N | R | R | S | S | L | A | E | P | R | Q | A | V | Q |
| ROP16.cma | Q | H | - | - | - | T | S | Q | K | T | C | L | K | R | G | A | L | D | V | W | A | L | G | L | A | I | F | E | F | V | C | F | R | L | P | Y | S | L | S | N | L | P | S | S | F | W | S | R | V | E |
| ROP17.cma | M | I | T | - | - | R | L | R | N | A | I | P | Y | T | P | Q | I | D | S | W | M | L | G | I | S | L | Y | R | L | W | C | G | N | F | P | F | G | M | T | L | D | A | T | - | A | L | Q | V | A | G |
| ROP18.cma | - | - | - | - | - | F | Q | A | T | G | I | T | Y | T | F | P | T | D | A | W | Q | L | G | I | T | L | Y | C | I | W | C | K | E | R | P | T | P | A | D | G | I | W | D | Y | L | H | - | - | - | - |
| ROP19-29-38.cma | S | P | - | - | - | Q | E | K | T | E | T | T | I | T | E | E | R | D | A | W | A | L | G | T | L | L | F | L | L | W | C | G | S | Y | P | F | F | S | D | E | Q | A | - | - | D | L | P | P | S | D |
| ROP20.cma | F | F | N | - | - | T | P | E | E | K | I | A | L | T | Q | V | V | D | S | W | S | L | G | M | A | A | W | E | V | L | C | M | S | E | P | F | D | G | M | R | F | S | E | D | D | L | Y | N | M | K |
| ROP21-27.cma | I | R | - | - | - | D | G | E | K | N | I | M | T | T | E | K | K | D | T | W | A | L | G | V | A | L | Y | K | L | W | C | R | Q | Y | P | F | E | M | G | E | L | W | - | - | S | K | D | A | F | D |
| ROP22.cma | V | H | E | H | G | E | E | V | G | P | L | E | A | H | P | T | V | D | A | W | R | L | A | V | I | L | Y | K | L | W | C | L | E | S | M | Y | E | R | S | S | Y | H | - | - | Q | F | V | A | R | Q |
| ROP23.cma | - | - | - | - | - | R | E | K | V | F | F | R | R | D | E | K | T | N | A | W | A | L | G | I | T | L | Y | Y | I | W | C | N | R | F | P | F | G | I | L | D | G | D | S | A | P | P | P | E | L | F |
| ROP24.cma | - | - | - | - | - | - | - | D | E | E | C | V | Y | S | Y | L | T | D | S | W | Q | L | G | L | T | L | Y | Q | I | W | C | L | R | L | P | F | G | L | M | T | P | V | V | A | D | D | P | D | Y | Y |
| ROP25.cma | A | L | K | N | G | K | - | - | - | - | M | S | M | G | P | E | R | D | S | W | A | V | G | V | S | A | Y | R | L | A | C | K | K | F | P | F | S | L | D | R | K | F | N | A | P | R | H | I | A | T |
| ROP26.cma | F | T | K | V | A | A | L | R | P | N | V | V | Y | G | Q | A | R | D | S | W | S | L | G | R | V | M | F | Q | L | L | C | G | Q | H | P | F | G | H | L | G | P | P | A | S | P | L | V | T | A | K |
| ROP2-8.cma | E | R | A | M | L | H | Q | H | H | P | T | L | M | T | F | S | F | D | T | W | T | L | G | L | A | I | Y | W | I | W | C | A | D | L | P | N | - | - | - | - | - | - | - | - | - | - | - | - | T | - |
| ROP28.cma | A | Y | R | Y | G | Y | Q | - | - | - | - | S | P | R | A | K | R | D | A | Y | A | L | G | V | T | F | Y | K | L | I | C | H | R | G | P | F | H | L | E | Q | L | E | H | E | A | L | H | M | L | S |
| ROP30.cma | F | L | R | D | D | E | D | - | F | S | V | V | Y | S | P | L | R | D | A | W | A | A | G | I | V | C | Y | Y | I | W | C | S | A | L | P | Y | N | L | N | Q | V | Y | N | R | G | K | Q | M | F | E |
| ROP31.cma | A | - | - | - | - | T | Q | F | S | Q | V | R | V | E | L | T | H | D | S | W | M | L | A | F | T | I | Y | F | L | W | C | G | R | L | P | W | G | I | A | I | E | E | G | D | Y | R | K | I | V | A |
| ROP32.cma | Y | F | A | - | - | D | P | D | R | R | L | D | L | L | A | S | R | D | S | W | C | L | G | V | V | F | F | K | L | W | C | H | R | L | P | F | G | L | Q | L | S | R | S | D | M | P | R | F | M | N |
| ROP33.cma | - | - | - | - | - | - | - | D | Q | I | I | L | N | T | A | K | S | D | V | Y | A | L | G | V | C | L | K | Q | L | A | K | R | - | Y | P | - | - | - | - | - | - | - | - | - | - | - | - | - | - | - |
| ROP34.cma | Y | L | L | R | K | G | - | Y | K | R | S | R | Y | G | E | K | T | D | V | Y | S | L | G | V | A | F | Q | H | L | A | F | M | G | L | G | V | - | - | - | - | - | - | - | - | - | - | - | - | - | - |
| ROP35.cma | - | - | - | - | - | - | - | T | D | W | V | H | F | T | E | K | S | D | V | Y | A | L | G | Q | C | F | K | R | M | L | K | Y | M | H | K | T | - | - | - | - | - | - | - | - | - | - | - | - | - | - |
| ROP36.cma | S | H | H | G | P | - | - | G | Q | T | F | E | A | S | F | A | L | D | A | W | R | L | G | V | S | L | Y | K | L | W | C | R | E | S | W | R | E | P | K | P | G | E | T | W | A | F | V | K | A | Q |
| ROP37.cma | L | F | - | - | - | G | L | G | T | Q | L | P | A | S | E | D | F | D | S | W | N | L | G | Q | L | L | F | S | L | L | C | G | K | L | P | W | A | L | G | Q | V | F | A | R | E | M | A | A | E | K |
| ROP39.cma | R | E | T | - | - | T | P | D | Q | E | V | T | L | A | F | S | Q | D | A | W | A | L | G | M | L | M | Y | D | I | F | C | H | N | W | P | F | M | G | M | D | Y | N | N | D | D | T | S | N | T | I |
| ROP40.cma | - | - | - | - | - | - | T | S | R | G | R | R | F | T | P | A | D | N | S | C | D | L | G | L | V | I | F | S | L | W | C | N | G | S | P | P | E | R | E | P | S | G | - | - | - | - | - | - | - | - |
| ROP41.cma | V | L | A | G | W | R | R | A | I | F | A | Q | V | T | E | A | A | D | L | W | A | L | G | M | T | L | Y | K | L | W | T | G | V | Y | P | F | G | L | S | S | K | H | F | R | T | K | D | V | T | M |
| ROP42-43-44.cma | - | - | - | - | - | L | E | H | P | E | T | P | Y | T | Y | S | R | D | A | Y | S | L | G | I | I | L | F | E | L | W | C | G | R | L | P | F | D | L | G | T | P | G | V | D | N | S | H | P | A | T |
| ROP45.cma | W | A | - | - | - | T | Q | Q | K | K | F | S | P | S | M | A | T | D | S | W | S | L | G | V | A | I | I | Q | L | W | C | G | E | T | P | F | R | V | P | N | A | L | W | N | P | V | A | V | A | Q |
| ROP46.cma | - | - | - | - | - | - | - | V | N | D | N | A | N | S | F | A | S | D | I | W | A | L | G | V | T | F | K | N | V | L | T | A | A | T | - | - | - | - | - | - | - | - | - | - | - | - | - | - | - | - |
| ROP4-7.cma | A | G | L | R | - | F | G | P | R | K | T | T | M | T | H | A | M | D | A | W | T | L | G | A | T | I | F | F | I | W | C | F | K | A | P | D | T | G | S | G | Y | E | Y | S | I | E | F | L | - | - |
| ROP47.cma | - | - | - | - | - | S | P | P | E | N | A | A | F | T | F | S | L | N | A | W | E | L | G | C | I | L | Y | Q | L | W | C | E | G | L | P | F | G | L | S | P | T | P | E | T | T | K | K | R | S | G |
| ROP48.cma | - | - | - | - | - | - | - | - | - | - | - | - | - | - | - | - | A | E | S | S | R | L | G | H | L | V | Y | Y | V | Y | T | G | K | T | F | T | K | F | W | K | F | R | C | T | D | N | V | L | - | - |
| ROP49.cma | - | - | - | - | - | - | - | - | - | M | G | V | G | G | A | K | Q | D | T | V | R | L | G | N | I | A | H | A | I | Y | T | G | K | Y | P | S | D | D | L | D | - | - | - | - | - | - | - | - | - | - |
| ROP50.cma | R | A | L | S | R | K | L | N | I | S | L | L | H | T | K | A | A | D | V | F | C | L | G | K | A | F | E | A | L | A | D | L | D | L | - | - | - | - | - | - | - | - | - | - | - | - | - | - | - | - |
| ROP5.cma | A | - | - | - | - | - | - | - | N | T | A | T | F | T | H | A | L | D | A | W | Q | L | G | L | S | I | Y | R | V | W | C | L | V | L | P | F | G | L | V | - | - | - | - | T | P | G | R | P | S | - |
| ROPK-Eten1.cma | F | I | R | K | G | E | - | - | - | - | T | N | I | S | H | K | Y | D | A | W | S | A | G | L | T | C | Y | V | L | M | T | N | R | L | P | Y | R | I | R | T | G | R | G | M | E | E | Y | L | A | M |
| ROPK-Eten2a.cma | I | L | K | G | E | R | R | A | V | P | V | I | S | H | A | K | G | D | M | W | S | L | G | V | I | I | Y | E | L | F | T | K | T | L | P | Y | G | L | T | E | P | A | N | I | A | A | A | L | G | S |
| ROPK-Eten2b.cma | I | R | K | G | I | E | T | A | T | P | V | V | P | E | A | R | S | D | L | W | S | L | G | A | L | L | Y | E | L | F | T | G | D | L | P | Y | G | L | S | A | P | A | H | S | M | D | R | L | Y | I |
| ROPK-Eten3.cma | S | R | R | S | - | - | - | G | E | G | L | C | P | E | A | N | S | N | L | W | S | L | G | V | L | L | F | E | L | Y | T | G | T | E | P | Y | G | R | V | E | G | S | T | W | G | E | R | A | W | H |
| ROPK-Eten4.cma | L | K | K | S | E | G | S | G | F | A | A | K | V | H | E | K | C | D | L | W | S | L | G | V | L | L | Y | E | L | F | S | G | R | Q | F | E | D | I | L | S | L | E | A | T | S | N | V | L | A | I |
| ROPK-Eten5.cma | A | T | R | K | S | E | G | A | S | H | P | V | V | D | E | K | S | D | M | W | G | L | G | I | C | L | Y | Q | I | F | T | G | K | M | P | Y | G | L | E | S | - | - | - | - | S | D | P | P | V | K |
| ROPK-Eten6.cma | L | W | C | Y | E | D | C | K | G | L | A | E | A | N | A | K | A | D | M | W | S | L | G | I | V | L | H | Q | I | L | M | D | E | L | P | F | G | L | S | D | I | P | S | - | D | E | S | A | I | P |
| ROPK-Unique.cma | G | A | R | N | P | E | G | - | - | E | V | M | A | T | P | A | M | D | S | W | S | L | G | V | T | L | F | E | L | W | C | N | K | L | P | F | G | I | S | E | P | R | G | G | V | G | H | N | I | R |
|  |  |  |  |  |  |  |  |  |  | 210 |  |  |  |  |  |  |  |  |  | 220 |  |  |  |  |  |  |  |  |  | 230 |  |  |  |  |  |  |  |  |  | 240 |  |  |  |  |  |  |  |  |  | 250 |

|  |  |  |  |  |  |  |  |  |  |  |  |  |  |  |  |  |  |  |  |  |  |  |  |  |  |  |  |  |  |  |  |  |  |  |  |  |  |  |  |  |  |  |  |  |  |  |  |  |  |  |
| --- | --- | --- | --- | --- | --- | --- | --- | --- | --- | --- | --- | --- | --- | --- | --- | --- | --- | --- | --- | --- | --- | --- | --- | --- | --- | --- | --- | --- | --- | --- | --- | --- | --- | --- | --- | --- | --- | --- | --- | --- | --- | --- | --- | --- | --- | --- | --- | --- | --- | --- |
|  |  |  |  |  |  |  |  |  |  |  |  |  |  |  |  |  | \* |  |  |  |  |  |  |  |  |  |  |  |  |  |  |  |  |  |  |  |  |  |  |  |  |  | \* |  |  |  |  |  |  | \* |
| ROPK.gtest | H | L | Q | R | L | V | R | S | G | K | N | T | D | F | S | R | C | K | M | D | M | P | E | R | V | Q | E | L | I | R | G | L | L | Q | P | D | P | E | K | R | L | T | P | L | Q | A | M | E | E | S |
| NTE.gtest | I | V | E | R | V | S | A | S | P | L | D | L | G | F | S | S | C | H | A | N | I | P | E | P | V | K | Q | L | I | Y | G | F | L | N | F | D | P | E | D | R | L | L | P | L | Q | A | M | E | - | T |
| ETEN.gtest | H | F | V | Q | L | L | R | E | T | A | N | V | D | E | M | D | V | K | M | G | V | P | R | R | W | R | E | L | I | V | R | L | L | E | T | D | R | S | K | R | I | S | A | E | E | V | A | A | E | Y |
| ROPKL-v-ROPK.gtest | - | - | - | - | - | - | - | - | - | - | - | - | - | - | - | - | - | - | - | R | R | P | E | M | L | D | D | L | S | Q | K | M | T | Q | A | D | P | E | K | R | P | T | I | E | E | V | M | E | - | D |
| BPK1.cma | - | - | - | - | - | - | - | - | - | - | - | - | - | - | - | - | - | - | - | R | Q | D | E | R | L | D | K | L | S | Q | K | M | I | E | E | E | P | G | N | R | P | T | I | E | E | I | M | K | - | D |
| ROP11.cma | V | L | A | Q | L | Q | A | D | F | I | A | A | N | F | T | R | C | R | H | H | V | N | E | R | F | Q | F | I | I | Q | S | L | L | D | V | N | P | Y | K | R | G | A | P | S | A | Q | L | T | - | A |
| ROP16.cma | H | L | S | R | L | R | L | S | D | F | S | V | K | - | - | D | C | N | - | E | S | D | P | A | V | M | G | I | V | A | Q | F | L | N | P | D | P | Q | E | R | P | E | L | P | K | F | V | N | - | S |
| ROP17.cma | I | V | I | R | S | S | A | S | S | L | D | - | - | F | A | S | C | H | - | D | I | P | E | Q | F | R | E | M | I | V | G | F | L | R | K | T | P | G | V | R | L | S | P | Q | Q | A | L | E | - | Q |
| ROP18.cma | - | - | - | - | - | - | - | - | - | - | - | - | - | F | A | D | C | P | - | S | T | P | E | L | V | Q | D | L | I | R | S | L | L | N | R | D | P | Q | K | R | M | L | P | L | Q | A | L | E | - | T |
| ROP19-29-38.cma | L | L | K | N | L | V | T | M | A | K | T | T | R | F | H | L | C | K | - | D | M | P | P | A | V | R | H | L | I | T | G | F | L | Q | W | N | S | D | D | R | R | R | P | G | D | V | V | D | - | D |
| ROP20.cma | V | I | A | A | L | P | E | G | G | D | D | T | R | A | L | R | C | R | G | R | P | P | A | P | L | R | K | A | V | E | C | L | L | D | R | N | A | A | T | R | C | K | A | L | D | L | F | K | T | S |
| ROP21-27.cma | R | V | A | E | V | S | R | D | E | L | N | F | Y | - | - | P | C | A | P | D | M | P | V | A | V | M | D | L | I | Q | Q | M | L | T | P | N | I | E | T | R | P | T | L | R | D | L | Y | V | S | H |
| ROP22.cma | D | L | Q | R | I | P | D | S | E | S | N | I | T | F | L | A | C | R | K | D | M | P | S | S | L | Q | R | L | L | R | Q | M | F | E | P | D | P | A | N | R | I | T | S | A | E | A | V | K | N | S |
| ROP23.cma | Q | P | V | P | P | E | F | G | - | - | - | - | - | - | - | V | C | V | H | N | M | P | D | P | V | K | V | L | V | A | E | L | L | D | R | Q | R | R | A | R | P | T | A | N | T | A | M | Q | - | R |
| ROP24.cma | V | V | E | E | V | L | R | K | P | D | A | E | L | S | F | G | C | V | A | N | I | P | E | Y | V | E | S | L | I | K | Q | L | L | Q | F | N | P | E | N | R | L | T | P | A | R | I | L | V | - | S |
| ROP25.cma | H | I | A | N | V | - | - | - | - | - | K | E | T | T | S | R | C | G | - | N | E | D | R | V | V | M | S | I | V | L | R | L | L | Q | P | D | P | S | K | R | A | T | V | D | Q | L | V | H | - | E |
| ROP26.cma | R | I | S | M | L | - | - | - | - | - | A | R | D | R | P | T | C | F | P | S | A | P | Q | D | L | M | S | I | M | A | N | F | L | K | F | D | A | N | E | R | P | T | P | L | E | I | V | R | R | Y |
| ROP2-8.cma | - | - | K | D | A | - | - | - | - | - | P | L | G | F | R | R | C | K | - | N | I | P | Q | P | V | R | A | L | L | E | G | F | L | R | Y | P | K | E | D | R | L | L | P | L | Q | A | M | E | - | T |
| ROP28.cma | G | I | S | R | F | R | R | K | D | W | V | E | R | - | - | E | C | P | - | I | A | D | E | S | L | L | T | I | V | K | L | L | L | D | P | Q | E | D | R | R | W | T | P | L | D | L | V | R | - | K |
| ROP30.cma | F | I | A | D | L | - | - | - | - | - | P | T | S | F | - | T | C | G | P | P | E | A | N | E | V | R | R | I | I | K | D | L | L | S | L | D | R | S | T | R | K | T | P | Q | C | I | V | E | Q | S |
| ROP31.cma | I | L | D | Q | N | T | P | E | T | L | N | F | H | - | - | N | C | N | - | G | I | P | A | G | I | Q | Q | L | I | R | L | L | L | A | K | D | P | N | R | R | W | T | P | E | K | A | I | A | E | L |
| ROP32.cma | Q | L | A | T | I | E | R | D | G | L | V | P | D | F | A | S | C | R | G | H | M | P | E | D | V | K | D | L | I | T | A | L | L | Y | Y | D | R | Y | S | R | E | A | P | L | S | L | I | E | K | S |
| ROP33.cma | - | - | - | - | - | - | - | - | - | - | - | - | - | - | - | - | - | - | - | K | T | A | D | K | L | Q | D | L | S | D | K | M | T | E | A | N | P | R | R | R | F | T | L | E | E | A | L | G | - | H |
| ROP34.cma | - | - | - | - | - | - | - | - | - | - | - | - | - | - | - | - | - | - | - | Q | V | P | T | Q | L | A | K | L | I | K | K | M | T | S | P | D | P | E | K | R | P | L | I | G | E | V | M | E | - | D |
| ROP35.cma | - | - | - | - | - | - | - | - | - | - | - | - | - | - | - | - | - | - | - | P | V | P | D | E | L | W | D | L | V | R | K | M | T | A | K | D | P | E | D | R | P | T | M | K | Q | V | M | E | - | H |
| ROP36.cma | Q | R | G | R | T | S | R | S | G | L | D | L | Q | - | - | H | C | R | A | D | T | P | S | F | I | L | R | L | I | Q | Q | F | L | E | P | N | P | E | T | R | L | T | P | I | D | A | V | K | G | T |
| ROP37.cma | F | Y | R | Q | F | I | K | E | V | K | R | E | K | F | A | F | C | P | G | G | T | K | L | Q | L | I | S | I | V | K | L | L | L | D | P | T | H | E | T | R | W | T | A | R | K | L | V | D | E | H |
| ROP39.cma | I | L | A | G | L | L | D | R | A | A | A | V | D | T | R | G | C | D | N | R | V | E | S | E | I | K | D | L | I | S | R | L | L | D | V | D | P | E | N | R | L | D | V | L | S | F | Y | M | H | S |
| ROP40.cma | - | W | V | N | L | D | - | - | - | - | - | - | - | F | S | S | C | E | S | E | V | P | V | L | V | Q | Q | I | I | C | G | L | C | R | A | H | G | Y | P | P | Q | N | A | F | Q | T | L | G | - | S |
| ROP41.cma | Y | I | Q | Q | L | R | E | D | K | S | W | P | D | F | Q | R | R | D | A | Q | V | H | P | E | V | Q | R | I | I | R | G | L | L | C | F | D | R | T | Q | R | L | R | L | S | E | V | V | R | - | T |
| ROP42-43-44.cma | L | Y | Q | T | V | R | N | L | D | Q | R | L | S | F | D | V | C | P | S | D | M | P | E | S | V | K | T | L | I | R | K | F | L | T | R | R | R | W | T | R | L | V | P | Q | A | A | L | R | - | D |
| ROP45.cma | Y | L | D | Q | L | L | R | R | T | Q | G | K | I | F | S | S | C | P | G | I | T | N | P | E | V | K | R | I | V | T | G | L | L | T | Y | D | H | V | K | R | W | L | P | L | D | I | L | F | T | S |
| ROP46.cma | - | - | - | - | - | - | - | - | - | - | - | - | - | - | - | - | - | - | - | V | R | A | E | V | L | D | G | L | S | N | R | M | T | A | S | D | P | A | A | R | P | S | V | D | D | V | L | L | - | D |
| ROP4-7.cma | - | - | - | - | - | - | - | - | - | - | - | - | - | F | S | R | C | - | - | R | R | A | E | N | V | K | L | L | V | Y | K | L | I | N | P | S | V | E | A | R | L | L | A | L | Q | A | T | E | - | T |
| ROP47.cma | L | A | G | T | S | T | R | P | V | K | H | L | V | F | T | G | C | V | K | N | M | P | V | E | M | R | Q | L | I | R | R | L | L | Q | E | D | P | H | K | R | L | L | P | T | E | I | L | R | - | D |
| ROP48.cma | - | - | - | - | - | - | - | - | - | - | - | - | - | F | N | D | V | N | V | L | A | D | K | L | I | R | N | A | I | K | A | L | I | T | C | N | K | D | K | R | I | L | P | Q | E | I | T | A | S | I |
| ROP49.cma | - | - | - | - | - | - | - | - | - | - | - | - | - | - | - | K | C | N | D | T | D | V | V | V | I | R | A | A | I | M | R | L | L | C | C | G | E | C | P | M | Q | K | P | A | D | M | V | V | A | S |
| ROP50.cma | - | - | - | - | - | - | - | - | - | - | - | - | - | - | - | - | - | - | - | E | N | G | L | M | L | E | V | L | V | E | R | M | T | Q | A | D | S | E | K | R | P | S | L | N | D | I | A | R | - | D |
| ROP5.cma | - | - | L | R | V | - | - | - | - | - | - | P | G | F | F | S | C | T | - | P | M | P | D | F | V | Q | T | L | I | R | R | F | L | N | F | D | R | R | R | R | L | L | P | L | E | A | M | E | - | T |
| ROPK-Eten1.cma | L | D | T | T | H | - | - | - | - | - | T | A | R | H | P | S | H | A | L | G | V | P | A | V | W | A | A | A | V | S | E | L | L | N | R | D | R | A | K | R | P | T | P | L | Q | L | L | D | R | Y |
| ROPK-Eten2a.cma | Y | F | G | K | L | I | E | K | D | I | A | A | D | M | L | I | P | E | M | R | V | P | Q | R | W | Q | E | L | L | V | K | L | L | Q | V | R | R | D | K | R | I | S | A | N | E | V | A | Q | E | Y |
| ROPK-Eten2b.cma | H | F | T | E | L | L | Q | R | D | I | S | S | A | V | L | V | P | E | M | H | V | P | E | R | W | Q | E | L | L | T | R | L | L | Q | V | K | R | E | K | R | I | T | A | D | E | V | I | K | E | F |
| ROPK-Eten3.cma | L | S | E | N | L | L | K | E | K | V | R | S | D | V | L | I | P | K | L | N | V | P | F | R | W | K | Q | L | I | L | R | L | L | E | P | R | R | S | E | R | I | T | A | W | E | I | I | R | E | F |
| ROPK-Eten4.cma | E | L | N | E | L | V | N | G | E | V | S | S | S | E | L | A | S | R | L | G | A | G | P | K | W | Q | Q | L | T | I | R | L | T | Q | P | S | R | D | K | R | I | S | S | E | E | I | F | N | Y | Y |
| ROPK-Eten5.cma | T | F | N | F | M | K | E | H | G | V | S | A | Q | S | L | R | G | R | L | G | V | P | V | R | W | Q | E | L | I | M | G | L | L | E | I | D | R | D | D | R | I | D | A | E | T | V | S | R | K | F |
| ROPK-Eten6.cma | H | V | V | Q | L | - | R | E | T | A | N | V | D | E | M | D | V | K | M | G | V | S | R | R | W | R | E | L | V | V | R | L | L | E | T | D | R | S | K | R | I | S | A | E | E | V | A | A | E | Y |
| ROPK-Unique.cma | S | L | Y | K | L | P | E | D | G | L | K | R | E | F | Q | R | C | R | A | M | V | P | E | R | W | Q | D | L | I | H | K | L | L | Q | P | Q | P | E | Q | R | I | T | A | L | Q | V | W | R | E | H |
|  |  |  |  |  |  |  |  |  |  | 260 |  |  |  |  |  |  |  |  |  | 270 |  |  |  |  |  |  |  |  |  | 280 |  |  |  |  |  |  |  |  |  | 290 |  |  |  |  |  |  |  |  |  | 300 |

|  |  |  |  |
| --- | --- | --- | --- |
|  |  | \* | \* |
| ROPK.gtest | P | F | F |
| NTE.gtest | P | E | F |
| ETEN.gtest | T | D | L |
| ROPKL-v-ROPK.gtest | P | F | F |
| BPK1.cma | P | L | F |
| ROP11.cma | Y | F | P |
| ROP16.cma | Y | T | F |
| ROP17.cma | F | S | L |
| ROP18.cma | A | A | F |
| ROP19-29-38.cma | F | L | A |
| ROP20.cma | P | L | F |
| ROP21-27.cma | P | V | F |
| ROP22.cma | S | F | F |
| ROP23.cma | E | I | F |
| ROP24.cma | R | D | F |
| ROP25.cma | P | F | F |
| ROP26.cma | P | Q | L |
| ROP2-8.cma | P | E | Y |
| ROP28.cma | V | P | F |
| ROP30.cma | F | L | Y |
| ROP31.cma | N | I | F |
| ROP32.cma | P | L | F |
| ROP33.cma | S | F | F |
| ROP34.cma | P | F | F |
| ROP35.cma | P | Y | F |
| ROP36.cma | S | L | F |
| ROP37.cma | P | F | F |
| ROP39.cma | D | F | F |
| ROP40.cma | H | E | F |
| ROP41.cma | S | Q | V |
| ROP42-43-44.cma | A | N | F |
| ROP45.cma | P | - | L |
| ROP46.cma | P | C | L |
| ROP4-7.cma | P | E | Y |
| ROP47.cma | P | V | F |
| ROP48.cma | P | F | F |
| ROP49.cma | P | L | F |
| ROP50.cma | K | F | F |
| ROP5.cma | P | E | F |
| ROPK-Eten1.cma | P | H | F |
| ROPK-Eten2a.cma | A | D | L |
| ROPK-Eten2b.cma | S | D | L |
| ROPK-Eten3.cma | P | D | L |
| ROPK-Eten4.cma | G | D | L |
| ROPK-Eten5.cma | Q | D | L |
| ROPK-Eten6.cma | T | D | L |
| ROPK-Unique.cma | P | D | F |
|  |  |  |  |
